# Matrix Gla Protein is a novel regulator of TGFβ–dependent fibroblast activation and kidney fibrosis

**DOI:** 10.1186/s43556-026-00518-0

**Published:** 2026-08-06

**Authors:** Jonatan Barrera-Chimal, Jocelyn López-Ramírez, Nathalie Henley, Peng Gao, Yeye Ma, Nicolas Desjardins-Lecavalier, Santiago Costantino, Virginie Royal, Stijn M. Agten, Teresa Borrás, Vincent Pichette, Darren A. Yuen, Leon J. Schurgers, Casimiro Gerarduzzi

**Affiliations:** 1 Centre de Recherche de l’Hôpital Maisonneuve-Rosemont, Centre Affilié à l’Université de Montréal, Montréal, Québec Canada; 2 Département de Médecine, Faculté de Médecine, Université de Montréal, Montréal, Québec Canada; 3 Département d’Ophtalmologie, Faculté de Médecine, Université de Montréal, Montréal, Québec Canada; 4 Department of Biochemistry, Cardiovascular Research Institute Maastricht (CARIM), Maastricht University, Maastricht, The Netherlands; 5 Department of Ophthalmology, University of North Carolina School of Medicine, Chapel Hill, NC USA; 6 Keenan Research Centre for Biomedical Science, Li Ka Shing Knowledge Institute, St. Michael’s Hospital (Unity Health Toronto) and Department of Medicine, University of Toronto, Toronto, ON Canada

**Keywords:** CKD, Fibroblast activation, TGFβ signaling, Smad3, BMP-2, Extracellular matrix

## Abstract

Kidney fibrosis is the pathologic hallmark of chronic kidney disease (CKD) and is driven by fibroblast-to-myofibroblast transformation (FMT), excessive extracellular matrix accumulation, and persistent activation of transforming growth factor-β (TGFβ) signaling. Although TGFβ is a central mediator of fibrosis, its pleiotropic physiological functions have limited the development of direct anti-fibrotic therapies, highlighting the need to identify context-specific regulators of this pathway. Matrix Gla Protein (MGP) is a vitamin K-dependent extracellular protein best known for inhibiting vascular calcification; however, its role in kidney fibrosis remains unknown. Here, we demonstrate that MGP expression is markedly upregulated in fibroblasts from fibrotic kidneys in mouse models of chronic kidney injury and in human CKD samples. Using inducible global and fibroblast-specific *Mgp* knockout mice, we show that *Mgp* deficiency attenuates renal fibrosis, suppresses fibroblast activation, and preserves kidney function following folic acid nephropathy and unilateral ureteral obstruction. Mechanistically, MGP promotes FMT and amplifies TGFβ receptor/Smad3 signaling, whereas genetic or molecular inhibition of MGP blunts TGFβ-driven fibroblast activation both in vivo and in vitro. We further identify phosphorylation and γ-carboxylation as essential post-translational modifications required for the profibrotic activity of MGP. In addition, MGP interacts with bone morphogenetic protein-2 (BMP-2), reduces BMP-2 protein abundance, and counteracts BMP-2-mediated suppression of TGFβ signaling, thereby enhancing profibrotic responses. Collectively, these findings identify MGP as a previously unrecognized regulator of TGFβ-dependent fibroblast activation and kidney fibrosis, supporting MGP as a potential fibroblast-associated target for future strategies aimed at limiting CKD progression.

**Supplementary Information:**

The online version contains supplementary material available at 10.1186/s43556-026-00518-0.

## Introduction

Chronic kidney disease (CKD) is a major global health burden affecting an estimated 11–13% of the population, corresponding to nearly 850 million individuals worldwide [1, 2]. Regardless of the initiating cause, kidney fibrosis represents the final common pathological pathway driving CKD progression and is characterized by fibroblast activation and excessive extracellular matrix (ECM) deposition [3–5]. Even though the remodeling of the ECM is required for tissue repair, this process can become maladaptive following severe or repetitive injury and lead to progressive fibrosis, ultimately causing organ dysfunction [6]. Transforming growth factor-β (TGFβ) signaling is a central driver of renal fibrogenesis, orchestrating fibroblast-to-myofibroblast transformation (FMT), promoting ECM production, and suppressing matrix degradation [7–9]. Following kidney injury, TGFβ activation leads to the accumulation of α-smooth muscle actin (αSMA)–positive myofibroblasts, the principal effector cells responsible for pathological matrix deposition [7, 8]. Despite recent therapeutic advances that slow CKD progression [10, 11], direct targeting of TGFβ signaling has proven challenging due to its pleiotropic roles in tissue homeostasis, immune regulation, and repair [5]. As a result, identifying novel context-specific modulators of TGFβ–driven fibrosis remains an important unmet clinical need [5].

Matrix Gla protein (MGP) is a small secreted protein traditionally recognized for its role in inhibiting vascular calcification [12, 13]. Under physiological conditions, MGP is predominantly produced by vascular smooth muscle cells and undergoes two major post-translational modifications: phosphorylation and γ-carboxylation [14]. Specifically, MGP contains three conserved serine phosphorylation sites (Ser-22, −25, −28) and five (four in mice) glutamic acid residues (Glu-21, −56, −60, −67 and −71) that can be carboxylated into γ-carboxyglutamic acid (Gla) residues by the enzyme γ-glutamylcarboxylase and its co-factor Vitamin K [14]. Carboxylation of MGP is essential for its ability to prevent vascular calcification by enabling calcium ion binding and inhibiting osteogenic differentiation [15, 16], whereas phosphorylation has been proposed to regulate its extracellular secretion [16]. In addition, MGP inhibits vascular calcification by binding bone morphogenetic protein-2 (BMP-2) and decreasing its osteogenic activity in vascular smooth muscle cells [17–19].

Although increased MGP expression has been reported in several fibrotic disorders, including Crohn’s disease [20], non-alcoholic steatohepatitis [21], vascular fibrosis and calcification [22], and kidney fibrosis [23], whether MGP actively contributes to fibrogenesis or simply reflects tissue remodeling remains unknown. In kidney disease, elevated MGP expression correlates with interstitial fibrosis and inflammation, but its functional role and underlying mechanisms have not been established [24]. Investigating the direct contribution of MGP to fibrosis has been challenging because global *Mgp*-knockout mice die within the first 6–8 weeks of birth due to arterial calcification and blood vessel rupture [12].

Therefore, we hypothesized that MGP is not merely a marker of kidney injury but an active regulator of renal fibrogenesis that promotes fibroblast activation and enhances profibrotic signaling. To test this hypothesis, we combined analyses of human kidney biopsies, single-cell transcriptomic datasets, mechanistic studies in cultured fibroblasts, and inducible global and fibroblast-specific *Mgp* knockout mouse models of kidney fibrosis. Our findings identify MGP as a previously unrecognized regulator of fibroblast-to-myofibroblast transformation and TGFβ-dependent fibrotic remodeling, providing new insight into the molecular mechanisms driving chronic kidney disease progression and highlighting MGP as a potential fibroblast-associated target for future antifibrotic strategies.

## Results

### MGP expression levels are increased in human and mouse fibrotic kidneys

**Fig. 1:**
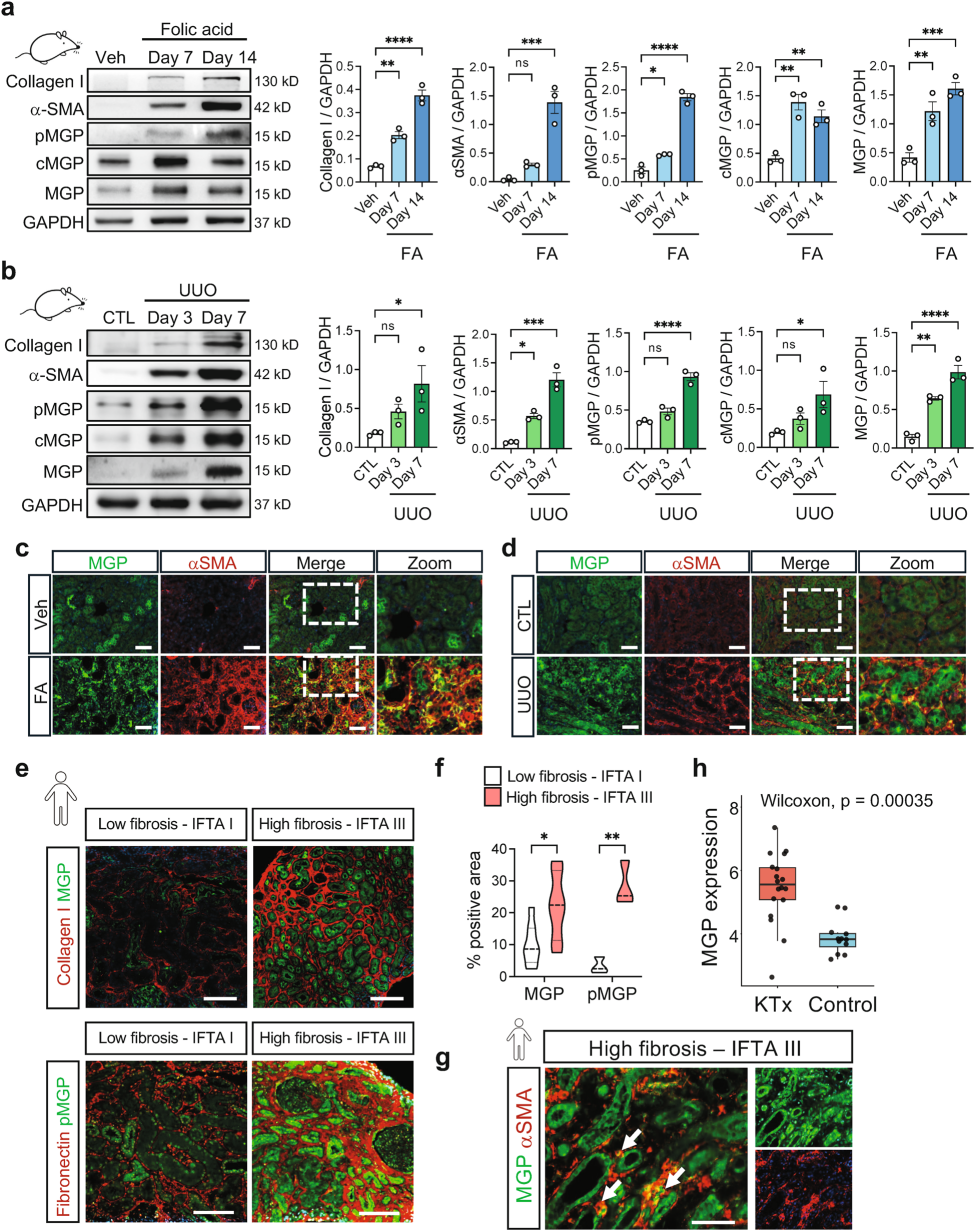
MGP upregulation in fibrotic kidneys of mouse models of fibrosis and kidney transplant patients. Western blot analysis was performed to evaluate the kidney levels of collagen I, αSMA, phospho-MGP (pMGP), carboxy-MGP (cMGP), total-MGP, and GAPDH following **a** 7 or 14 days of folic acid (FA) injection or **b** unilateral ureteral obstruction (UUO) for 3 or 7 days. Representative blot images are shown, and the graphs represent the densitometric analysis with normalization to GAPDH as a loading control (n = 3 per group). **c** and **d** Immunofluorescence of kidneys from **c** FA and **d** UUO mice showing αSMA (α-smooth muscle actin, red), total-MGP (green) and its co-localization (yellow) (*n* = 5) **e** Immunofluorescence of kidney transplant patient biopsies showing in the upper inset, collagen I (red) and total-MGP (green) and in the lower inset, fibronectin (red) and pMGP (green) staining in patients with mild fibrosis (interstitial fibrosis and tubular atrophy score (IFTA) I (n = 5) or in patients with severe fibrosis (n = 5, IFTA III). **f** Quantification of total-MGP and pMGP positive areas. **g** Immunofluorescence of kidney transplant patient biopsy showing αSMA (red), total-MGP (green) and its co-localization (yellow, indicated with arrows). Scale bar: 100 µm. **h** Comparison of MGP expression between kidney transplant patients (KTx) and normal groups using Wilcoxon rank-sum test; RNA-sequencing data were obtained from GEO dataset GSE135327. Data are presented as mean ± SEM and were analyzed using a one-way ANOVA followed by Dunnett’s post-hoc test (**a**, **b**) or Student’s t-test **f**. *p < 0.05, **p < 0.01, ***p < 0.001 and ****p < 0.0001

To investigate whether MGP levels are upregulated in kidney fibrosis, we determined the MGP protein levels in the commonly used mouse models of folic acid (FA) and unilateral ureteral obstruction (UUO)-induced kidney fibrosis. We found that renal total-MGP protein expression and its phosphorylated (phospho-MGP) and carboxylated (carboxy-MGP) forms are increased in both models of kidney fibrosis, and their upregulation correlated with the appearance of the fibrotic marker collagen I and myofibroblast activation marker αSMA (Fig. 1a and b). Moreover, through immunofluorescence analysis, we found that in the FA and UUO fibrosis models, MGP co-localizes with αSMA (Fig. 1c and d). To evaluate whether MGP expression is increased in human CKD, immunostaining for total-MGP and phospho-MGP was performed in kidney biopsies from kidney transplant patients with severe fibrosis (interstitial fibrosis and tubular atrophy grade III (IFTA III)) or in biopsies with minimal fibrosis (interstitial fibrosis and tubular atrophy grade I (IFTA I)). Increased total- and phospho-MGP staining was observed in fibrotic kidneys (IFTA III), correlating with elevated collagen I and fibronectin levels (Figs. 1e and 1f). Similarly, MGP co-localized with αSMA in human samples (Fig. 1g). In addition, we found increased *MGP* mRNA levels in RNA-sequencing data from 18 kidney transplant patients with fibrosis [25] compared with control samples (Fig. 1h). Overall, these data indicate that MGP expression is increased in mouse and human fibrotic kidney disease.

### Inducible deletion of *Mgp* protects against kidney fibrosis in mice

**Fig. 2:**
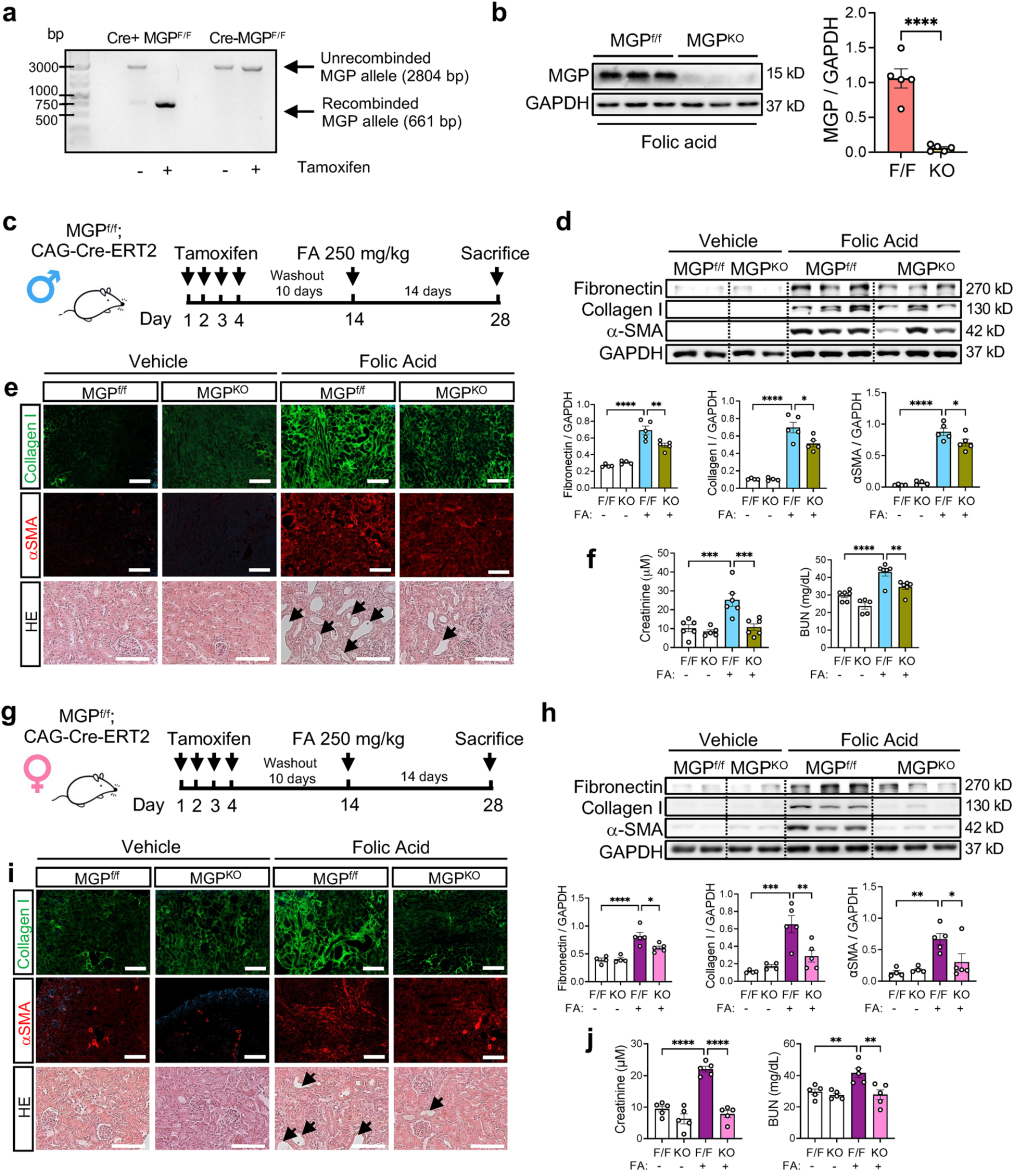
Inducible and ubiquitous *Mgp* knockout protects against kidney fibrosis. **a** Genotyping showing the amplification of the unrecombined *Mgp* allele prior to the tamoxifen administration on Cre^−^ and Cre^+^ mice, and the amplification of the recombined allele in the Cre^+^ mice following tamoxifen administration. **b** Western blot analysis to evaluate the levels of MGP and GAPDH in MGP^f/f^ and MGP^KO^ mice post-FA administration and confirming MGP absence. **c** Experimental design; male mice were injected with tamoxifen for four consecutive days, and after a 10-day washout period, they received a single folic acid injection (250 mg/kg). The mice were sacrificed at day 14 post-FA injection. **d** Western blot analysis showing the levels of fibronectin, collagen I, αSMA (α-smooth muscle actin), and GAPDH. Upper insets are representative blots, and the graphs depict the densitometric analyses (Veh MGP^F/F^ *n* = 4, Veh MGP^KO^ *n* = 4, FA MGP^F/F^ *n* = 5, FA MGP^KO^ *n* = 5). **e** Immunofluorescence images of kidneys from MGP^f/f^ or MGP^KO^ mice following vehicle or FA injections showing collagen I (green) or αSMA (red) and representative images of H&E staining. **f** Plasma creatinine and urea were determined (Veh MGP^F/F^ *n* = 6, Veh MGP^KO^ *n* = 5, FA MGP^F/F^ *n* = 6, FA MGP^KO^ *n* = 6). **g** Experimental design; female mice were injected with tamoxifen for four consecutive days, and after a 10-day washout period, they received a single folic acid injection (250 mg/kg). The mice were sacrificed at day 14 post-FA injection. **h** Western blot analysis was performed to evaluate the kidney levels of fibronectin, collagen I, αSMA, and GAPDH. Upper insets are representative blots, and the graphs depict the densitometric analyses (Veh MGP^F/F^ *n* = 4, Veh MGP^KO^ *n* = 4, FA MGP^F/F^ *n* = 5, FA MGP^KO^ *n* = 5). **i** Immunofluorescence images of kidneys from MGP^f/f^ or MGP^KO^ mice following vehicle or FA injections showing collagen I (green) or αSMA (red) and representative images of H&E staining. **j** Plasma creatinine and urea were determined (Veh MGP^F/F^ *n* = 5, Veh MGP^KO^ *n* = 5, FA MGP^F/F^ *n* = 5, FA MGP^KO^ *n* = 5). Data are presented as mean ± SEM and were analyzed using Student’s t-test (b) or a one-way ANOVA followed by Šídák post-hoc test (**d**, **f**, **h**, **j**). *p < 0.05, **p < 0.01, ***p < 0.001 and ****p < 0.0001. Images in panels **e** and **i** are representative of five mice per group. Scale bar: 100 µm

We next sought to investigate whether *Mgp* deletion impacts kidney fibrosis development. Since *Mgp*-knockout mice die within two months after birth due to arterial calcification and aortic rupture [12], we generated a mouse model of inducible and ubiquitous deletion of *Mgp* (MGP^KO^) by crossing *Mgp* floxed (MGP^f/f^) mice with mice expressing the tamoxifen-inducible Cre under the chicken β-actin promoter (Fig. S1). The allele recombination and *Mgp* deletion were confirmed by genotyping (Fig. 2a) and western blot (Fig. 2b). Following tamoxifen-induced recombination of *Mgp*, mice were challenged with the FA-induced kidney fibrosis model (Fig. 2c and Fig. S2a). Male mice injected with FA developed kidney fibrosis as evidenced by an augmented expression and interstitial accumulation of fibronectin, collagen I and αSMA at days 7 and 14 post-FA injection, whereas in the MGP^KO^ mice these alterations were attenuated (Fig. 2d-e and Fig. S2b-c). In addition, we detected structural injury and renal dysfunction in MGP^f/f^ mice receiving FA as shown by tubular dilation and an increase in plasma creatinine and urea levels, effects that were reduced in MGP^KO^ mice (Fig. 2e-f and Fig. S2c-d). Moreover, in MGP^f/f^ female mice, kidney injury was evident at day 14 post-FA injection as shown by increased levels of fibrotic markers, tubular dilation and renal dysfunction (Fig. 2g-j). In contrast, female MGP^KO^ mice showed a more pronounced protective phenotype than that observed in males (Fig. 2g-j). We also evaluated the effect of *Mgp* deletion on kidney fibrosis induced by UUO, a more severe and rapidly progressive model. *Mgp* deletion was confirmed in this model (Fig. S3a). Despite the increased fibrotic pressure, female mice with widespread *Mgp* deletion exhibited partial protection, as evidenced by reduced levels of fibrotic markers and attenuated structural damage (Fig. S3). No changes in renal function were observed in the UUO model due to the compensation of the undamaged contralateral kidney (Fig. S3e). Altogether, these findings indicate an important role for MGP in kidney fibrosis development.

### MGP promotes fibroblast-to-myofibroblast transformation

**Fig. 3:**
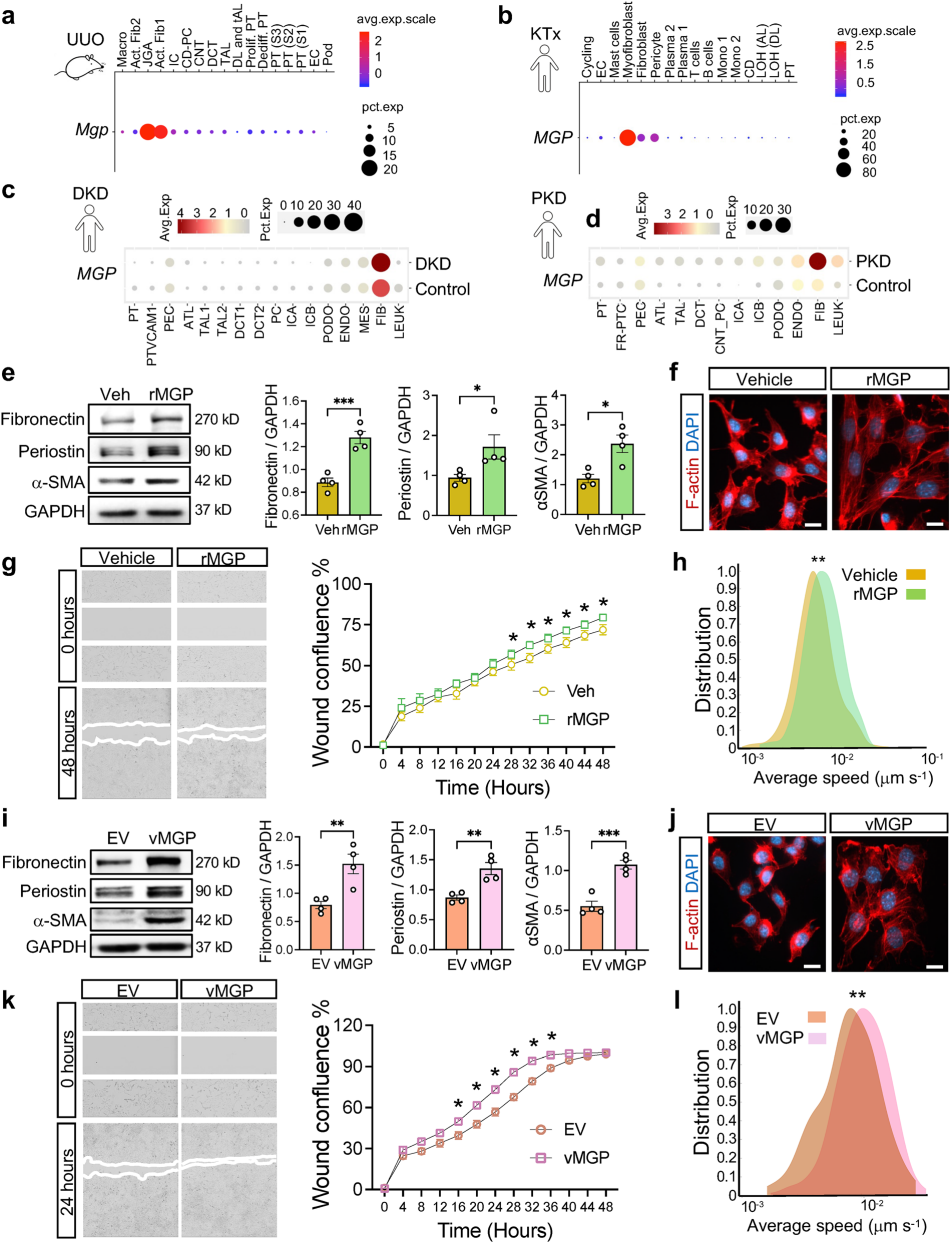
MGP is upregulated in fibroblasts and induces fibroblast-to-myofibroblast transformation. **a** *Mgp* mRNA levels in different cell subsets as detected by single-nucleus RNA sequencing in the mouse model of UUO-14 days. *MGP* mRNA levels in different cell subsets detected by single-cell RNA sequencing of **b** human rejecting kidney transplant biopsy (KTx), **c** human diabetic kidney disease (DKD), and **d** autosomal dominant polycystic kidney disease (PKD). **a** to **d** were generated using the http://humphreyslab.com/SingleCell/ website. **e** NIH3T3 cells were treated with recombinant MGP protein (rMGP), 50 ng/mL and 24 h after the cell lysates were analyzed by western blot to evaluate the levels of fibronectin, periostin, αSMA (α-smooth muscle actin), and GAPDH. Representative blots are shown, and the graphs depict the densitometric analyses (*n* = 4 per group). **f** Phalloidin-Rhodamine staining was performed 24 h after rMGP treatment to assess for stress fiber formation. Scale bar: 10 µm. Images are representative of three independent experiments. **g** NIH3T3 cells were plated to form a monolayer, and a scratch-wound was performed to evaluate the migration capacity of the cells when rMGP was added compared with vehicle. Representative images of four independent experiments at baseline (0 h) and 48 h are shown and the wound confluence % that was monitored every 4 h. **h** The average speed of NIH3T3 cells was tracked with traction force microscopy following 24 h of rMGP stimulation. **i** NIH3T3 cells were transfected with Myc-*MGP*-expressing vector (vMGP) or empty vector (EV) and 24 h after the cell lysates were analyzed by western blot to evaluate the levels of fibronectin, periostin, αSMA, and GAPDH. Representative blots are shown, and the graphs depict the densitometric analyses (*n* = 4 per group). **j** Phalloidin-Rhodamine staining was performed 24 h after vMGP or EV transfection to assess for stress fiber formation. Scale bar: 10 µm. Images are representative of three independent experiments. **k** NIH3T3 cells were plated to form a monolayer, and a scratch-wound was performed to evaluate the migration capacity of vMGP transfected cells versus EV. Representative images of four independent experiments at 0 and 24 h are shown and the wound confluence % that was monitored every 4 h. **l** The average speed of NIH3T3 cells was tracked with traction force microscopy following 24 h after vMGP or EV transfection. Data are presented as mean ± SEM and were analyzed using an unpaired, two-tailed Student’s t-test (**e, i, g, k**). *p < 0.05, **p < 0.01 and ***p < 0.001

Since *Mgp* knockout mice were protected against kidney fibrosis, we further investigated through which cellular responses MGP could mediate its fibrotic effects. First, we explored the cellular localization of *MGP* expression by datamining existing single-cell RNA sequencing (RNA-seq) datasets from mouse UUO, human rejecting kidney allograft biopsies, patients with diabetic kidney disease and polycystic kidney disease [26–29]. We found that under these fibrotic conditions, *Mgp/MGP* mRNA levels are mainly detected in activated fibroblasts/myofibroblasts (Fig. 3a-d). Further analysis using single-cell/single nucleus RNA-seq datasets showed that in mouse polycystic kidney disease [30] and ischemia/reperfusion [31] models, *Mgp* is mainly found in fibroblast/interstitial cells (Fig. S4). These analyses support our observation showing MGP co-localization with αSMA (myofibroblast marker) in our mouse models of FA and UUO kidney fibrosis (Fig. 1c and d) and in kidney biopsies from kidney transplant patients (Fig. 1g). Since excessive myofibroblast transformation is a key feature in kidney fibrosis, we performed in vitro studies to evaluate the potential of MGP to stimulate fibroblast-to-myofibroblast transformation (FMT). We show that a 24-h treatment with recombinant MGP protein can induce FMT in NIH3T3 fibroblasts, as shown by the increased expression of the myofibroblast markers αSMA, periostin and fibronectin (Fig. 3e). Moreover, the actin cytoskeleton was reorganized, as evidenced by the formation of F-actin stress fibers, a hallmark of FMT (Fig. 3f). A wound-healing assay revealed that MGP accelerated the myofibroblast-like migratory response of fibroblasts compared with vehicle treatment (Fig. 3g). We further evaluated how MGP influences the mechanical properties of fibroblasts using traction force microscopy, revealing that recombinant MGP treatment enhanced the average speed (µm s^−1^) of the NIH3T3 fibroblasts (Fig. 3h). Similarly, transfection of an *MGP*-Myc-expressing vector confirmed an effect of MGP on the induction of myofibroblast markers (Fig. 3i), stress fiber formation (Fig. 3j), accelerated migration (Fig. 3k) and increased average speed of fibroblasts (Fig. 3l) as compared with empty-vector-transfected cells.

Since the phospho-MGP and carboxy-MGP forms were upregulated in the kidneys of mice and patients with fibrotic kidney disease, we investigated whether these post-translational modifications are required for MGP-mediated induction of FMT. We first generated MGP mutant versions in which each of the serine phosphorylation sites (S22, S25 and S28) were individually mutated to alanine and created a triple serine-to-alanine mutant (S3XA) (Fig. S5a). As expected, wild-type (WT)-MGP upregulated the expression of fibronectin, periostin and αSMA, promoted stress fiber formation and accelerated wound healing (Figs. S5b-e and S6). Each of the individual phospho-null mutants and the triple mutant versions of MGP were unable to induce FMT in NIH3T3 fibroblasts, confirming the crucial role of MGP phosphorylation in this process (Figs. S5b-e and S6). We also investigated the effect of mutating each of the carboxylation glutamic acid sites (E21, E56, E60, E67 and E71) to glycine. Each individual mutant as well as a mutant carrying three carboxylation site mutations (Fig. S7a) failed to induce FMT in NIH3T3 fibroblasts, unlike WT MGP, showing the crucial effect of MGP carboxylation in promoting FMT (Figs. S6 and S7b-e). Of note, the lack of fibrotic effects upon transfection of the mutant versions of MGP was not due to defects in MGP expression levels (Figs. S5b and S7b) nor to defects in MGP secretion since MGP-phosphorylation mutants were found at higher levels in the medium compared with the WT, whereas no changes were observed for the carboxylation mutants (Fig. S8). Altogether, our data confirm that MGP can induce fibroblast activation, and that phosphorylation and carboxylation are required for MGP to induce fibrotic effects in fibroblasts.

### Inducible and specific deletion of *Mgp* in fibroblasts protects against kidney fibrosis in mice

**Fig. 4:**
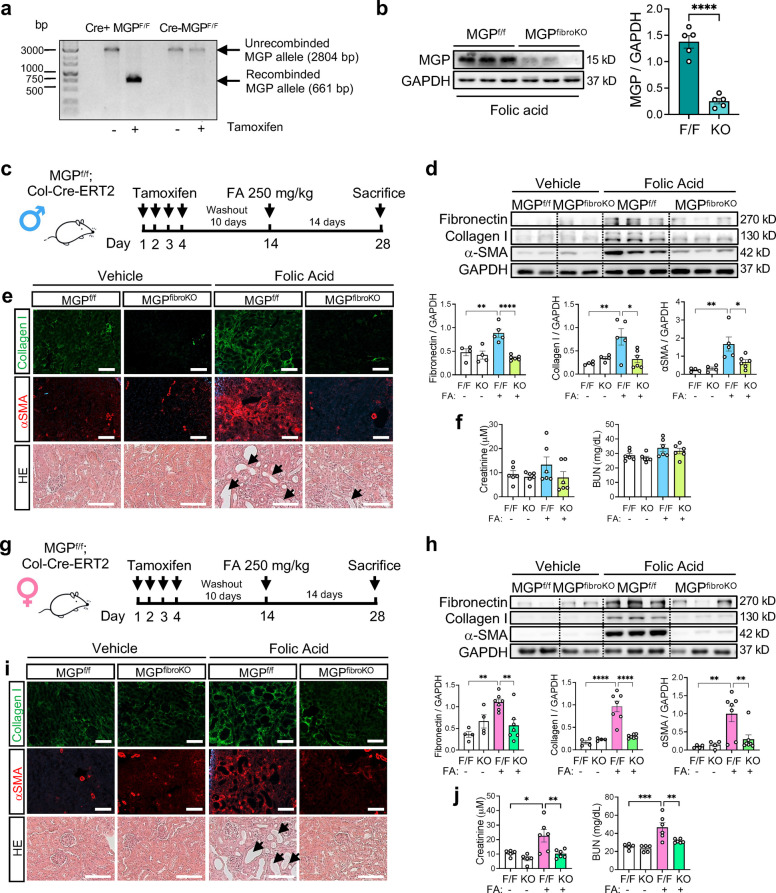
Fibroblast-specific *Mgp* knockout protects against FA-induced kidney fibrosis. **a** Genotyping showing the amplification of the unrecombined *Mgp* allele prior to the tamoxifen administration on Cre^−^ and Cre^+^ mice, and the amplification of the recombined allele in the Cre^+^ mice following tamoxifen administration. **b** Western blot analysis was performed in kidney tissue lysates from mice treated with FA to induce kidney fibrosis and to evaluate the kidney levels of MGP and GAPDH. **c** Experimental design; male mice were injected with tamoxifen for four consecutive days, and after a 10-day washout period, they received a single folic acid injection (250 mg/kg). The mice were sacrificed at day 14 post-FA injection. **d** Western blot analysis showing the levels of fibronectin, collagen I, αSMA (α-smooth muscle actin), and GAPDH. Upper insets are representative blots and the graphs depict the densitometric analyses (Veh MGP^F/F^ *n* = 4, Veh MGP^fibroKO^ *n* = 4, FA MGP^F/F^ *n* = 5, FA MGP^fibroKO^ *n* = 6). **e** Immunofluorescence images of kidneys from MGP^f/f^ or MGP^fibroKO^ mice following vehicle or FA injections showing collagen I (green) or αSMA (red) and representative images of H&E staining. **f** Plasma creatinine and urea were determined (Veh MGP^F/F^ *n* = 6, Veh MGP^fibroKO^ *n* = 6, FA MGP^F/F^ *n* = 6, FA MGP^fibroKO^ *n* = 6). **g** Experimental design; female mice were injected with tamoxifen for four consecutive days, and after a 10-day washout period, they received a single folic acid injection (250 mg/kg). The mice were sacrificed at day 14 post-FA injection. **h** Western blot analysis was performed to evaluate the kidney levels of fibronectin, collagen I, αSMA, and GAPDH. Upper insets are representative blots and the graphs depict the densitometric analyses (Veh MGP^F/F^ *n* = 4, Veh MGP^fibroKO^ *n* = 4, FA MGP^F/F^ *n* = 7, FA MGP^fibroKO^ n = 7). **i** Immunofluorescence images of kidneys from MGP^f/f^ or MGP^fibroKO^ mice following vehicle or FA injections showing collagen I (green) or αSMA (red) and representative images of H&E staining. **j** Plasma creatinine and urea were determined (Veh MGP^F/F^ *n* = 5, Veh MGP^fibroKO^ *n* = 5, FA MGP^F/F^ *n* = 6, FA MGP^fibroKO^ *n* = 6). Data are presented as mean ± SEM and were analyzed using Student’s t-test (**b**) or one-way ANOVA followed by Šídák post-hoc test (**d**, **f**, **h**, **j**). *p < 0.05, **p < 0.01, ***p < 0.001 and ****p < 0.0001. Images are representative of n = 5 mice per group **e**, **i**. Scale bar: 100 µm

Given that MGP showed significant fibrotic effects by promoting fibroblast activation (Fig. 3), and that *Mgp* upregulation is primarily observed in fibroblasts/myofibroblasts in several single-cell RNA-seq analyses (Fig. 3a-d and Fig. S4), we aimed to investigate whether fibroblast-specific deletion of *Mgp* (by breeding *Collagen1a2*-Cre with MGP^f/f^ mice, Fig. S9) could recapitulate the protective effect against kidney fibrosis observed with inducible ubiquitous *Mgp* deletion. Following tamoxifen-induced recombination of *Mgp* in fibroblast-specific knockout mice (MGP^fibroKO^, Fig. 4a), male and female mice were exposed to FA injury. MGP expression was drastically reduced in MGP^fibroKO^ mice exposed to FA (Fig. 4b). Male MGP^f/f^ mice developed extensive kidney fibrosis at days 7 and 14 as shown by increased expression of the fibrotic markers fibronectin, collagen I and αSMA, as well as interstitial deposition of collagen I and myofibroblasts (αSMA-positive interstitial cells; Fig. 4c-e and Fig. S10). Of note, mice lacking *Mgp* specifically in fibroblasts (MGP^fibroKO^) were protected against renal fibrotic alterations induced by FA. Moreover, extensive tubular dilation and increased creatinine and urea plasma levels were also observed in MGP^f/f^ mice receiving FA, effects that were prevented by fibroblast-specific *Mgp* deletion (Fig. 4e-f and Fig. S10). A similar pattern of FA-induced kidney fibrosis and renal dysfunction was observed in female MGP^f/f^ mice, while these effects were prevented in MGP^fibroKO^ mice 14 days after FA injection (Fig. 4g-j).

**Fig. 5:**
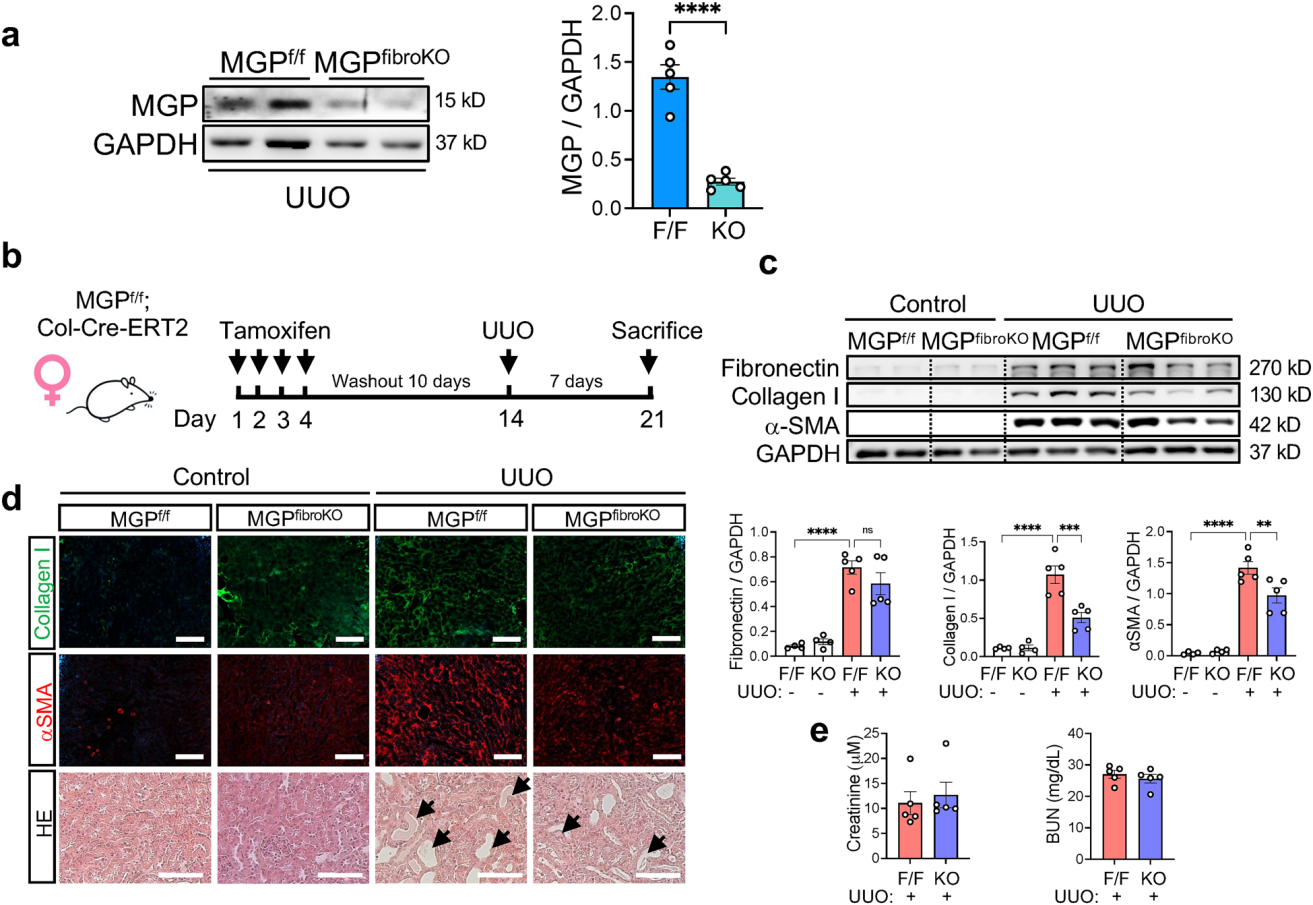
Fibroblast-specific *Mgp* knockout protects against UUO-induced kidney fibrosis. **a** Western blot analysis was performed in kidney tissue lysates from mice that underwent UUO to induce kidney fibrosis and to evaluate the kidney levels of MGP and GAPDH. **b** Experimental design; female mice were injected with tamoxifen for four consecutive days, and after a 10-day washout period, underwent UUO surgery. The mice were sacrificed at day 7 post-UUO. **c** Western blot analysis showing the levels of fibronectin, collagen I, αSMA (α-smooth muscle actin), and GAPDH. Upper insets are representative blots and the graphs depict the densitometric analyses (CTL MGP^F/F^ *n* = 4, CTL MGP^fibroKO^ *n* = 4, UUO MGP^F/F^ *n* = 5, UUO MGP^fibroKO^ *n* = 5). **d** Immunofluorescence images of kidneys from MGP^f/f^ or MGP^fibroKO^ mice following control or UUO surgery showing collagen I (green) or αSMA (red) and representative images of H&E staining. **e** Plasma creatinine and urea were determined (UUO MGP^F/F^ *n* = 5, UUO MGP^fibroKO^ *n* = 5). Data are presented as mean ± SEM and were analyzed using Student’s t-test (**a**) or one-way ANOVA followed by Šídák post-hoc test (**c**, **e**). *p < 0.05, **p < 0.01, ***p < 0.001 and ****p < 0.0001. Images are representative of n = 5 mice per group. Scale bar: 100 µm

Regarding the UUO-induced kidney fibrosis model, MGP inhibition was confirmed in MGP^fibroKO^ mice (Fig. 5a). Female MGP^f/f^ mice showed increased fibrotic marker levels at day 7 post-UUO, and a protective effect was observed in mice lacking *Mgp* in fibroblasts in which the levels of collagen I and αSMA were reduced (Fig. 5b-d). No changes in plasma creatinine or urea were observed, as expected with a functional contralateral kidney (Fig. 5e).

To address whether inducible *Mgp* deletion could lead to vascular calcification, both whole-body and fibroblast-specific *Mgp* knockout mice were administered tamoxifen to induce *Mgp* recombination at 8 weeks of age and were followed for 3 months after recombination to assess renal arterial calcification by Von Kossa staining. We did not detect evidence of arterial calcification in either inducible whole-body or fibroblast-specific *Mgp* knockout mice after 3 months of *Mgp* deletion (Fig. S11).

In summary, our findings suggest that MGP upregulation specifically in fibroblasts plays a key role in kidney fibrosis development. In addition, adult *Mgp* deletion was not associated with detectable renal arterial calcification by Von Kossa staining within the 3-month period examined.

### *Mgp* deficiency reduces TGFβ signaling in vitro and in vivo

**Fig. 6:**
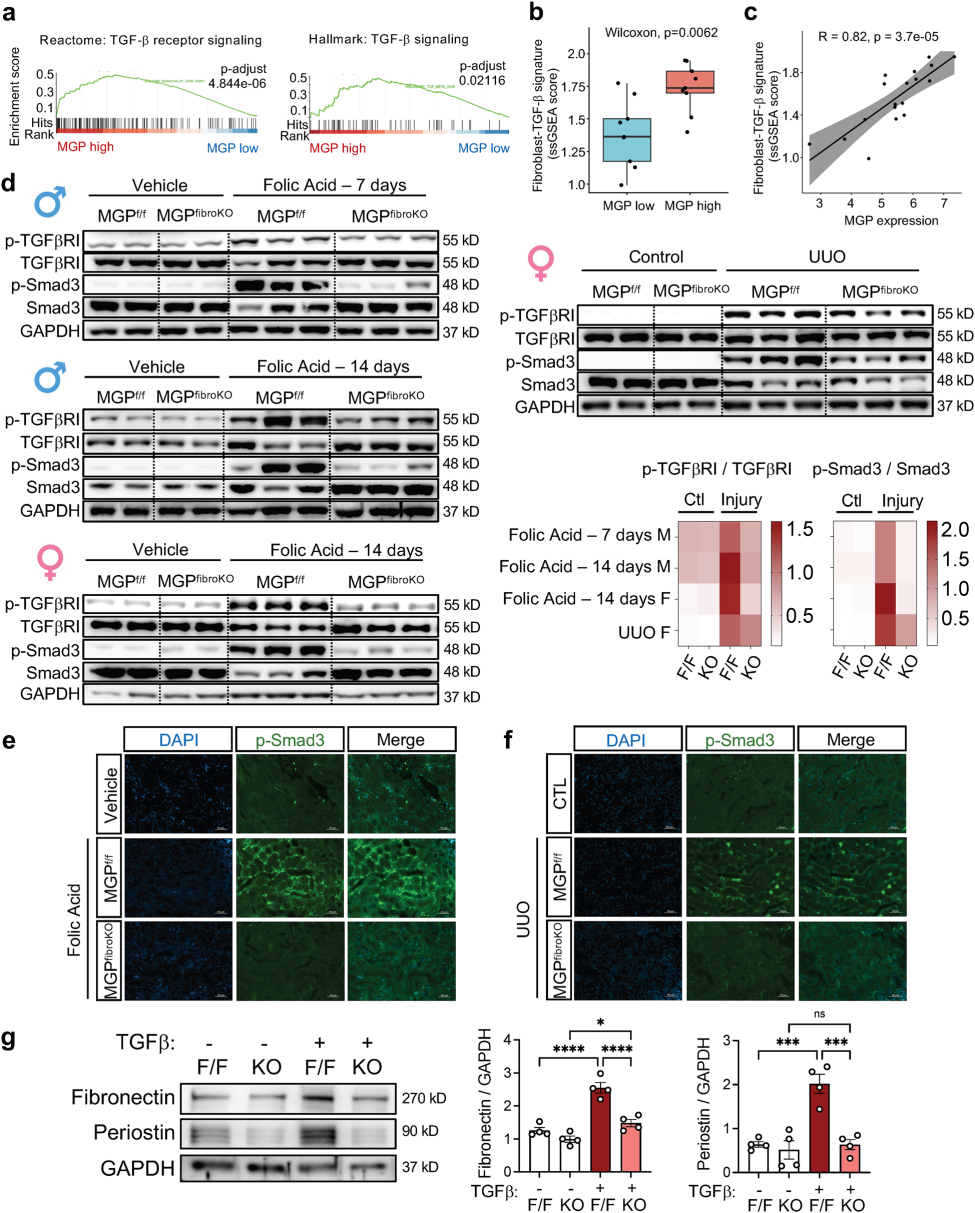
*MGP* expression is associated with TGFβ signaling signatures. **a** Gene set enrichment analysis (GSEA) of TGFβ–related pathways in *MGP*-high versus *MGP*-low kidney transplant patient samples with median-based stratification (Data from GEO dataset GSE135327). **b** Comparison of single-sample GSEA (ssGSEA) enrichment scores between *MGP*-high and *MGP*-low kidney transplant patient samples for the fibroblast–TGFβ gene signature analysis. **c** Correlation between *MGP* expression levels and fibroblast–TGFβ signature scores. **d** Western blot analysis was performed in kidney tissue lysates from the different cohorts of male and female mice who received FA injection or underwent UUO surgery to evaluate the kidney levels of phospho-TGFβRI (TGFβ receptor I), TGFβRI, phospho-Smad3, Smad3 and GAPDH. Representative blot images for each of the mouse cohorts are shown, and the heatmaps represent the densitometric analysis (For FA cohorts: Veh MGP^F/F^ n = 4, Veh MGP^fibroKO^ *n* = 4, FA MGP^F/F^ *n* = 5, FA MGP^fibroKO^ *n* = 5/For UUO cohort: CTL MGP^F/F^ n = 4, CTL MGP^fibroKO^ *n* = 4, UUO MGP^F/F^ *n* = 5, UUO MGP^fibroKO^ *n* = 5). **e** Immunofluorescence images of kidneys from MGP^f/f^ or MGP^fibroKO^ mice after 14 days of vehicle or FA injections showing nuclei (blue), phospho-Smad3 (green) or the merged image. **f** Immunofluorescence images of kidneys from MGP^f/f^ or MGP^fibroKO^ mice after 7 days of UUO showing nuclei (blue), phospho-Smad3 (green) or the merged image. Images are representative of n = 5 mice per group. Scale bar: 100 µm. **g** Primary fibroblasts isolated from kidneys of MGP^f/f^ or MGP.^fibroKO^ mice were treated with TGFβ (5 ng/mL), and 24 h later, the cell lysates were analyzed by western blot to evaluate the levels of fibronectin, periostin, and αSMA (α-smooth muscle actin). Representative blots are shown, and the graphs depict the densitometric analyses (*n* = 4 per group). Data are presented as mean ± SEM and were analyzed using a one-way ANOVA followed by Šídák post-hoc test (**g**). *p < 0.05, **p < 0.01, ***p < 0.001 and ****p < 0.0001

During kidney fibrotic remodeling, TGFβ signaling plays a key role in myofibroblast transformation and excessive extracellular matrix deposition [32, 33]. To investigate the relationship between MGP and TGFβ signaling, we used the RNA-seq data from kidney transplant patients exhibiting fibrosis (GSE135327) [25] and we stratified the samples by *MGP* expression levels. Gene set enrichment analysis (GSEA) revealed a significant enrichment of TGFβ signaling pathways in *MGP*-high compared to *MGP*-low samples using both median-based (Figs. 6a-b) and tertile-based stratification (Fig. S12). Furthermore, single-sample GSEA (ssGSEA) analysis using a fibroblast–TGFβ gene signature showed that *MGP*-high samples exhibited higher fibroblast–TGFβ signature enrichment scores than *MGP*-low samples (Fig. 6b). *MGP* expression was positively correlated with the TGFβ signature enrichment scores across disease samples (Fig. 6c). We then sought to investigate whether TGFβ signaling and TGFβ-elicited FMT/fibrotic phenotype were affected by MGP inhibition. We assessed the phosphorylation status of TGFβ receptor I (TGFβRI) and its downstream effector, Smad3, in male and female mice subjected to either FA- or UUO-induced injury. In both models of kidney fibrosis, TGFβRI and Smad3 phosphorylation were significantly increased in MGP^f/f^ mice, whereas reduced phosphorylation of both TGFβRI and Smad3 was observed in MGP^fibroKO^ kidneys compared with MGP^f/f^ controls following FA and UUO injury (Fig. 6d). Immunofluorescence demonstrated decreased interstitial p-Smad3-positive cells in the fibrotic kidneys from both FA (Fig. 6e) and UUO (Fig. 6f) models. Similarly, ubiquitous deletion of *Mgp* was associated with decreased TGFβRI and Smad3 phosphorylation following exposure to FA or UUO surgery (Fig. S13). We then isolated primary fibroblasts from MGP^f/f^ and MGP^fibroKO^ mice and stimulated them with TGFβ. As expected, primary fibroblasts isolated from WT mice increased fibronectin and periostin production upon TGFβ stimulation (Fig. 6g). In contrast, fibroblasts obtained from MGP^fibroKO^ mice were unable to upregulate fibrotic markers following TGFβ exposure (Fig. 6g).

**Fig. 7:**
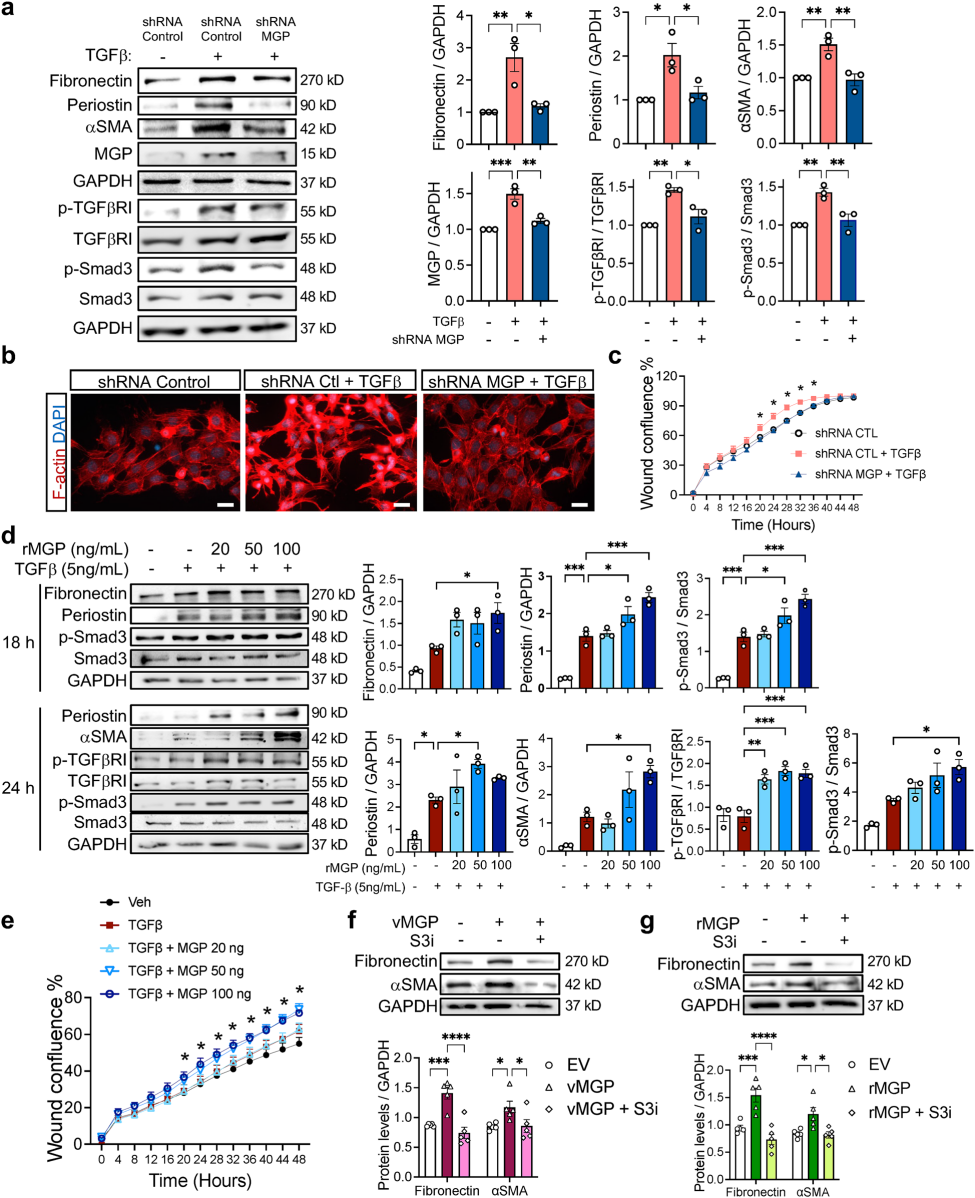
MGP inhibition reduced TGFβ-elicited FMT and signaling in vitro. **a** NIH3T3 cells were transfected with a plasmid expressing an shRNA against *Mgp* (shMGP) or a control (Ctl) shRNA. After 24 h, they were treated with TGFβ (5 ng/mL), and 24 h later, the cell lysates were analyzed by western blot to evaluate the levels of fibronectin, periostin, αSMA (α-smooth muscle actin), phospho-TGFβRI (TGFβ receptor I), TGFβRI, phospho-Smad3, Smad3, MGP and GAPDH. Representative blots are shown, and the graphs depict the densitometric analyses (*n* = 3 per group). **b** Phalloidin-Rhodamine staining was performed to assess for stress fiber formation. Scale bar: 10 µm. Representative images of at least three independent replicates are shown. **c** NIH3T3 cells were plated to form a monolayer, and a scratch-wound was performed to evaluate the migration capacity of TGFβ-stimulated cells with or without *Mgp* inhibition through shRNA transfection. The wound confluence % that was monitored every 4 h is represented in the graph (*n* = 4 per group). **d** NIH3T3 cells were treated with TGFβ (5 ng/mL) with or without increasing concentrations of recombinant MGP protein, and 18 or 24 h later, the cell lysates were analyzed by western blot to evaluate the levels of fibronectin, periostin, αSMA, phospho-TGFβRI, TGFβRI, phospho-Smad3, Smad3 and GAPDH. Representative blots are shown, and the graphs depict the densitometric analyses (*n* = 3 per group). **e** NIH3T3 cells were plated to form a monolayer, and a scratch-wound was performed to evaluate the migration capacity after stimulation with TGFβ and increasing concentrations of recombinant MGP. The wound confluence % that was monitored every 4 h (*n* = 4 per group). **f** NIH3T3 cells were pre-treated for 12 h with the Smad3 inhibitor (S3i; SIS3 1 µM) and then transfected with *MGP*-expressing vector. After 24 h, the cell lysates were analyzed by western blot to evaluate the levels of fibronectin, αSMA, and GAPDH. **g** NIH3T3 cells were pre-treated for 12 h with the Smad3 inhibitor (S3i; SIS3 1 µM) and then treated with recombinant MGP protein. After 24 h, the cell lysates were analyzed by western blot to evaluate the levels of fibronectin, αSMA, and GAPDH. Data are presented as mean ± SEM and were analyzed using a one-way ANOVA followed by Šídák post-hoc test (**a**, **c**, **d**, **e**, **f**, **g**). *p < 0.05, **p < 0.01, ***p < 0.001 and ****p < 0.0001

Next, we explored whether short hairpin RNA (shRNA)-*Mgp* inhibition could prevent the TGFβ-elicited FMT phenotype in NIH3T3 fibroblasts. As expected, TGFβ treatment induced an increase in the fibroblast activation markers fibronectin, periostin and αSMA, which aligned with increased MGP expression and phosphorylation of TGFβRI and Smad3 (Fig. 7a). Interestingly, silencing *Mgp* expression hindered TGFβ–induced fibroblast-to-myofibroblast transition (FMT), which was accompanied by reduced TGFβ receptor I levels and decreased Smad3 phosphorylation upon *Mgp* inhibition (Fig. 7a). In addition, TGFβ induced an increase in the F-actin staining and distribution into stress fibers that was prevented by *Mgp* downregulation (Fig. 7b). The scratch-wound assay revealed that although TGFβ treatment enhanced the migration capacity of NIH3T3 fibroblasts, inhibition of *Mgp* completely blunted this effect (Fig. 7c and Fig. S14a). When NIH3T3 cells were co-treated with TGFβ and increasing doses of MGP, it was observed that MGP enhanced the TGFβ-mediated FMT as evidenced by the augmented levels of fibronectin, periostin and p-Smad3 after 18 h and periostin, αSMA, p-TGFβRI and p-Smad3 after 24 h of co-treatment compared with TGFβ alone (Fig. 7d). An accelerated healing capacity of the fibroblasts co-treated with 50 and 100 ng/mL of MGP was observed compared with TGFβ treatment alone (Fig. 7e and Fig. S14b). A similar cytoskeletal rearrangement was observed with MGP and TGFβ co-treatments at 24 h (Fig. S15). To confirm that MGP effects are mediated through the TGFβRI/Smad3 axis, we stimulated NIH3T3 cells with MGP and the Smad3 inhibitor, SIS3. Smad3 inhibition blunted the FMT effects of MGP overexpression or recombinant protein treatment (Fig. 7f and g).

**Fig. 8:**
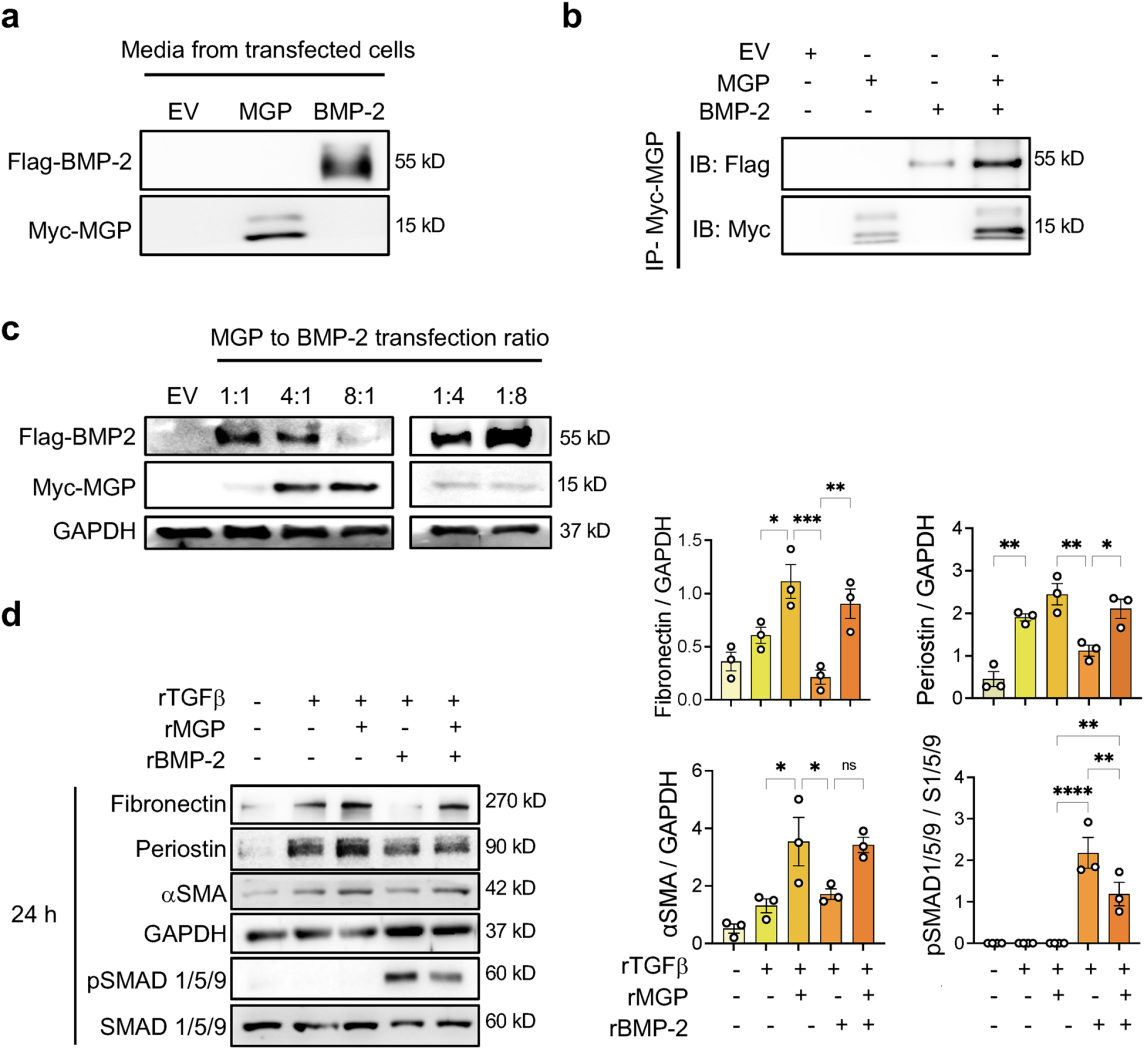
MGP interacts with BMP-2 and mitigates BMP-2-mediated suppression of TGFβ–induced fibroblast activation. **a** Western blot showing the detection of Flag-*BMP-2* and Myc-*MGP* in the medium from transfected NIH3T3 cells. **b** Co-immunoprecipitation analysis of media from Flag-BMP-2 and myc-MGP-transfected NIH3T3 fibroblasts representing the immunoblots (IB) against Flag (BMP-2) and Myc (MGP) following Myc immunoprecipitation, showing a physical interaction between secreted MGP and BMP-2. Immunoprecipitation of Myc-MGP resulted in the co-immunoprecipitation of Flag-BMP-2, indicating extracellular complex formation. **c** Increasing MGP expression by transfecting higher ratios of *MGP* vector vs *BMP-2* vector led to a dose-dependent reduction in BMP-2 protein levels, whereas increasing *BMP-2* amounts had no effect on MGP expression. Empty vector was added accordingly to maintain the total amount of transfected DNA constant. **d** NIH3T3 cells were treated with TGFβ (5 ng/mL), and/or recombinant MGP protein (50 ng/mL) and/or recombinant BMP-2 (50 ng/mL), and 24 h later, the cell lysates were analyzed by western blot to evaluate the levels of fibronectin, periostin, αSMA (α-smooth muscle actin), phospho-Smad1/5/9, Smad1/5/9 and GAPDH. Representative blots are shown, and the graphs depict the densitometric analyses (*n* = 3 per group). Co-treatment with MGP restored TGFβ–induced fibroblast activation and fibrotic marker expression, indicating that MGP counteracts the antifibrotic effects of BMP-2. Data are presented as mean ± SEM and were analyzed using a one-way ANOVA followed by Šídák post-hoc test (**c**). *p < 0.05, **p < 0.01, ***p < 0.001 and ****p < 0.0001

Given that MGP has been shown to modulate cellular signaling by binding and inhibiting BMP-2 in vascular smooth muscle cells [19, 34, 35], we next investigated whether a similar mechanism operates in fibroblasts to influence fibrotic signaling. BMP-2 has been reported to exert antifibrotic effects in kidney fibrosis [36, 37], raising the possibility that MGP may promote fibrosis by antagonizing BMP-2 activity and thereby relieving its inhibitory influence on TGFβ signaling. To test this hypothesis, we examined whether MGP can interact with BMP-2 and modulate its effects on TGFβ–induced fibroblast activation. Through immunoprecipitation studies in media derived from fibroblasts overexpressing BMP-2 and MGP (Fig. 8a), we found that MGP physically interacts with BMP-2 (Fig. 8b). Moreover, we show that increasing amounts of *MGP* overexpression led to a reduction in BMP-2 protein levels whereas increasing the amounts of *BMP-2* had no effect on MGP expression (Fig. 8c). Functionally, BMP-2 suppressed TGFβ1–induced FMT, whereas the presence of MGP restored TGFβ1–driven pathway activation despite BMP-2 exposure (Fig. 8d). Overall, these findings identify MGP as a potential regulator of profibrotic TGFβ signaling during kidney fibrotic remodeling. MGP expression was strongly associated with activation of TGFβ-related pathways in human datasets. Consistently, MGP deficiency suppressed TGFβ receptor signaling, Smad3 activation, and fibroblast-to-myofibroblast transition. In addition, we showed that MGP suppresses BMP-2 antifibrotic signaling. These data support a role for MGP in inducing fibroblast activation by promoting TGFβ signaling, ultimately leading to renal fibrosis progression.

## Discussion

MGP is a small protein, primarily known to be produced and secreted by vascular smooth muscle cells, where it locally acts to prevent vascular calcification. Although MGP expression has been reported to be increased in different pathological states [20–24], little is known about its involvement in disease pathogenesis. In the present study, we show that MGP protein expression and its phosphorylated and carboxylated forms are upregulated in mouse models of kidney fibrosis, as well as in fibrotic kidneys from kidney transplant patients. This observation is in agreement with previous RNA-seq studies in which, although this was not explicitly reported, reanalysis of publicly available datasets reveals a consistent upregulation of *Mgp* mRNA across multiple mouse models of CKD of different etiologies, including FA [38], *Collagen4a3*-KO mice [39], *mitochondrial transcription factor A*-KO mice [40] and mice with podocyte-specific overexpression of the *Apolipoprotein L1 (APOL1) G1/G2* risk variants [41] versus their respective controls. Specifically, single-cell and single-nucleus RNA-seq showed that *Mgp/MGP* mRNA upregulation in chronic nephropathies, such as mouse models of fibrosis as well as in human CKD, occurs primarily in fibroblasts/myofibroblasts. This transcript-level enrichment was corroborated by our immunofluorescence analyses showing MGP localization within the interstitial compartment and co-localization with αSMA-positive cells, implicating MGP in fibrogenic processes during CKD progression. The consistent restriction of *Mgp/MGP* mRNA to fibroblasts across several diseased kidney single-cell RNA-seq datasets supports fibroblasts as the dominant source of MGP in diseased kidneys. Accordingly, the epithelial MGP signal observed by immunofluorescence most likely represents extracellular deposition of fibroblast-derived protein within the tubular compartment.

A previous study reported that *Mgp*-null mice develop spontaneous damage of normal kidney structures, including endothelial alterations such as reduced capillary density and a slight impairment of renal function [42]. When *Mgp* heterozygous KO mice were subjected to UUO, they developed enhanced peritubular capillary rarefaction compared with WT mice [42]. In addition, *Mgp* KO mice showed increased tubular injury at baseline and 2 days following folic acid injection compared with WT; nevertheless, protection against kidney fibrosis was observed [43]. Since *Mgp*-knockout mice have vascular defects that lead to early mortality [12], the spontaneous kidney damage and enhanced capillary rarefaction observed after UUO could be associated with vascular calcification during development. To circumvent any developmental alterations due to a lack of MGP and to evaluate the specific role of MGP during kidney fibrosis, we developed two inducible models of *Mgp* deficiency, either widespread or fibroblast-specific. This allowed us to investigate if MGP could actively participate in fibrosis pathogenesis independently of its role in development and vascular calcification. Using these complementary genetic approaches, our data indicate that inducible deletion of *Mgp*, either systemically or specifically in fibroblasts, attenuates fibrotic remodeling and preserves kidney function in both folic acid and UUO-induced models of renal fibrosis. These protective effects were observed in both male and female mice, underscoring the robustness of the phenotype, although the protection appeared more pronounced in females, consistent with the lower susceptibility of female mice to kidney fibrosis in experimental models generally associated with sex hormone–mediated modulation of inflammatory and fibrotic pathways [44]. Moreover, our observation that fibroblast-specific knockout phenocopies whole-body knockout reinforces the major contribution of fibroblast MGP upregulation in fibrosis development.

While MGP upregulation in CKD has traditionally been interpreted as a compensatory response to mineral imbalance and calcification stress, our findings suggest a previously underappreciated role for MGP in kidney fibrotic remodeling. Specifically, we hypothesize that MGP may exert context- and time-dependent effects during CKD progression, where transient induction could participate in adaptive tissue repair while sustained expression contributes to maladaptive fibroblast activation and fibrosis progression. However, because *Mgp* deletion was induced prior to injury in the current study, this concept remains speculative and should be directly evaluated in therapeutic intervention models. Mechanistically, our in vitro studies show that recombinant MGP and *MGP* overexpression are sufficient to induce FMT in NIH3T3 cells, as evidenced by increased expression of myofibroblast markers, actin cytoskeleton reorganization and increased migration capacity. These findings are consistent with previous reports in glioblastoma, where inhibition of MGP reduced cell migration, suggesting a broader role for MGP in regulating mesenchymal cell behavior [45]. Importantly, we show that the profibrotic effects of MGP are blunted when its phosphorylation and γ-glutamyl carboxylation sites are mutated. Although phosphorylation has been proposed to regulate MGP secretion, and carboxylation is known to be essential for its anti-calcification function [46], the role of these post-translational modifications in fibrogenesis had not been previously explored. Of note, we show that MGP mutant versions lacking phosphorylation or carboxylation sites were secreted normally into the culture medium; secretion of the phosphorylation-site mutants was even increased relative to WT MGP. These findings support the hypothesis that phosphorylation modulates MGP trafficking and secretion [16], and highlight the need for further studies to elucidate the mechanisms regulating MGP trafficking. Although these data show that phosphorylation and carboxylation are required for MGP-mediated FMT, whether these modifications directly regulate MGP/BMP-2 binding remains to be determined and will require dedicated binding assays using defined MGP modification states and mutant variants. Future studies will also be required to determine whether pharmacological modulation of MGP expression or its post-translational modifications can be leveraged to therapeutically attenuate kidney fibrosis in CKD.

Our data show that MGP is actively involved in fibrosis pathogenesis by modulating fibroblast activation and promoting the secretion of extracellular matrix proteins. The fibrotic properties of MGP reported herein are consistent with previous reports showing that MGP could also promote liver fibrosis through its actions on hepatic stellate cells, in which it stimulates TGFβ signaling and Smad2/3 phosphorylation [21]. TGFβ1 is a multifunctional protein that participates in a variety of cellular functions, including cell growth, differentiation, FMT and ECM production [47], but is pathologically regarded as the most important profibrotic factor in fibrosis development in multiple organs [33, 47]. In the kidney, it is well recognized that TGFβ1 induces fibrosis via activation of the TGFβRI-dependent signaling pathways. Indeed, TGFβRI inhibition blunted kidney fibrosis in a rat model of UUO [48]. TGFβRI-dependent SMAD2 and SMAD3 phosphorylation have also been described in kidney fibrosis [49–51]. However, SMAD3 appears to be the predominant profibrotic factor since its deletion prevented kidney fibrosis [52–54], whereas mice deficient in SMAD2 develop an excessive fibrotic phenotype in the same model [55]. In this context, our findings indicate that MGP deficiency markedly suppresses profibrotic TGFβ signaling in vivo, as evidenced by reduced activation of TGFβRI and Smad3 during kidney fibrosis. Consistently, our in vitro data show that MGP is required for TGFβ–induced FMT in fibroblasts, positioning MGP as a previously unrecognized modulator of the TGFβ–Smad3 axis in renal fibrogenesis. In addition to directly enhancing TGFβ signaling, our findings suggest that MGP may further amplify fibrotic responses by modulating the balance between profibrotic and antifibrotic signaling pathways. MGP has been previously shown to bind and inhibit BMP-2 in vascular smooth muscle cells, and BMP-2 has been reported to exert antifibrotic effects in kidney fibrosis [36, 37]. Consistent with this concept, we show that MGP interacts with BMP-2, reduces its protein levels, and functionally counteracts BMP-2-mediated suppression of TGFβ1–induced FMT. These findings suggest that MGP promotes fibrotic signaling, at least in part, by sequestering and functionally neutralizing BMP-2, thereby removing an endogenous brake on TGFβ–mediated fibroblast activation. Although our data support a role for BMP-2 antagonism in mediating MGP-dependent fibroblast activation, additional signaling pathways may contribute to the profibrotic effects of MGP and warrant further investigation.

One limitation of the present study is the preventive nature of the genetic approach to inhibit *Mgp* which does not directly model therapeutic intervention after fibrosis establishment. Future studies will be required to determine whether delayed or stage-specific inhibition of MGP can attenuate established CKD progression while preserving potential early adaptive responses. Another important translational consideration is the established role of MGP as a critical inhibitor of vascular calcification [12, 13]. Because constitutive global *Mgp* deficiency causes severe arterial calcification and premature lethality, systemic inhibition of MGP could represent a potential safety concern, particularly in CKD patients who are already highly susceptible to cardiovascular disease and vascular calcification. Notably, inducible deletion of *Mgp* in adulthood did not result in detectable renal arterial calcification within the timeframe examined in this study (Fig. S11), unlike the calcification observed in 6–8-week-old germline whole-body *Mgp* knockout mice, suggesting important differences between developmental and adult *Mgp* loss. Nevertheless, the long-term cardiovascular safety of systemic MGP inhibition remains to be determined. Future therapeutic strategies may therefore need to focus on selectively targeting the pathological upregulation of MGP and its fibrotic pathway within renal fibroblasts while preserving its vascular protective functions. Another limitation is that the human data are primarily correlative and based on relatively small cohorts of kidney transplant biopsies. Therefore, future studies in larger and more diverse CKD populations will be required to determine whether MGP expression predicts disease progression or therapeutic response.

In summary, we show that MGP expression levels are increased in fibrotic kidneys during CKD. Through widespread or fibroblast-specific and inducible genetic deletion of *Mgp* and in vitro studies, our findings suggest a novel role for MGP in kidney fibrosis progression by promoting fibroblast-to-myofibroblast transformation and TGFβ signaling. In addition, our data suggest that MGP may function as an extracellular regulator of fibroblast activation and TGFβ signaling during kidney fibrotic remodeling. By linking MGP to BMP-2/TGFβ signaling crosstalk and fibroblast-to-myofibroblast transformation, this work expands the biological functions previously attributed to MGP beyond vascular calcification and provides a framework for future studies investigating MGP-directed antifibrotic therapies.

## Materials and methods

### Animal models

For the initial studies performed in WT mice, we used male C57BL/6J mice purchased from The Jackson Laboratory. Next, mice with inducible and ubiquitous deletion of *Mgp* were generated by crossing *Mgp*-floxed (MGP^f/f^) mice (previously generated and characterized by Dr. Teresa Borrás, North Carolina, USA [56]) with transgenic mice expressing the tamoxifen-inducible Cre recombinase driven by the chicken β-actin promoter/enhancer coupled with the cytomegalovirus (CMV) immediate-early enhancer (The Jackson Laboratory, Strain No. 004682). Mice with specific and inducible deletion of *Mgp* in fibroblasts were generated by crossing MGP^f/f^ mice with transgenic mice expressing the tamoxifen-inducible Cre recombinase driven by the *Col1a2* promoter (The Jackson Laboratory, Strain No. 029567). Mice were maintained in the Maisonneuve-Rosemont Hospital Research Center animal facility and fed Harlan Teklad rodent diet (#2018 Envigo, Canada) and water ad libitum. All experiments were conducted according to the Canadian Council on Animal Care guidelines for the care and use of laboratory animals and under the supervision and approval of our local animal care committee, *Comité de protection des animaux du Centre Intégré Universitaire de Santé et de Services Sociaux* (CIUSSS) *de l'Est-de-l'île-de-Montréal* (Approved Protocol No. 2022–2895), in compliance with the Animal Research: Reporting of in vivo Experiments (ARRIVE) Guidelines [57]. At 8–10 weeks of age, male and female mice from each transgenic mouse line were injected with tamoxifen diluted in corn oil (100 mg/kg). A specific set of mice was followed up for 3 months after tamoxifen injection to evaluate vascular calcification. In all the other mice, following a 10-day washout period, mice were randomized to receive a single intraperitoneal injection of folic acid (250 mg/kg in sodium bicarbonate) or vehicle and were studied after 7 or 14 days. Another set of mice underwent UUO surgery as previously described [58]. Briefly, following isoflurane anesthesia and butorphanol (2 mg/kg) analgesia, an incision was made in the left dorsal side of the mice, and the ureter was dissected and ligated with 4–0 sutures, the muscle and skin were sutured, and the mice were studied after 3 or 7 days post-UUO. WT C57BL/6 J mice were similarly injected with folic acid or subjected to UUO surgery. On the day of sacrifice, mice were anesthetized with isoflurane, a blood sample was taken by cardiac puncture to obtain the plasma and the kidneys were harvested. For the FA injury, one kidney was fixed in 10% paraformaldehyde for histological analyses, and the other kidney was flash-frozen in liquid nitrogen for quantitative polymerase chain reaction (qPCR) and Western blot analyses. For the UUO mice, the contralateral kidney (non-ligated ureter) was used as control tissue. Half of each kidney was used for histological studies and the other half for molecular analyses. Plasma creatinine and urea were determined using the commercially available kits: mouse creatinine assay kit (Crystal Chem No. 80350) and QuantiChrom™ Urea Assay Kit (BioAssay Systems) following the manufacturer's protocol. We routinely performed genotyping of the mice at endpoint to confirm *Mgp* allele recombination post-tamoxifen administration. All animals were included. Experiments were conducted without blinding during data acquisition; however, quantitative analyses were performed using predefined criteria and standardized pipelines to minimize bias.

### Protein extraction and western blotting

Total protein extracts were prepared from kidney samples or cells homogenized in radioimmunoprecipitation assay (RIPA) buffer (ThermoFisher Scientific) containing a cocktail of protease and phosphatase inhibitors (Roche Diagnostics). The extracts were centrifuged at 13,000 rpm for 10 min at 4 °C, the supernatant was collected, and the proteins were quantified using the Bicinchoninic Acid (BCA) protein assay (ThermoFisher Scientific). Following the quantification, 20–40 µg of protein were denatured for 10 min at 95 °C in Laemmli buffer and were subjected to electrophoresis on 9 to 12% Sodium Dodecyl Sulfate Polyacrylamide Gel Electrophoresis (SDS-PAGE) gels and transferred onto 0.22 µm Polyvinylidene fluoride (PVDF) membranes (BioRad). The membranes were blocked in 5% non-fat milk in Tris-buffered saline-Tween (TBS-T) for 90 min and incubated overnight at 4 °C with the following primary antibodies: anti-fibronectin (Abcam, ab23750, 1:10,000) anti-Collagen I (SouthernBiotech, 1310–01, 1:500), anti-αSMA (Millipore Sigma, A2547, 1:10,000), anti-phosphoMGP (Kind gift of Dr. Schurgers, Maastricht, The Netherlands, 1:750), anti-carboxyMGP [15] (Kind gift of Dr. Schurgers, Maastricht, The Netherlands, 1:750), anti-MGP (Proteintech, 10,734–1-AP, 1:1200), anti-periostin (Santa Cruz Biotechnology, sc-39863, 1:500), anti-myc (Cell Signaling, 22,765, 1:5000), anti-phosphoTGFβRI (ThermoFisher Scientific, PA5-40,298, 1:4000), anti-TGFβRI (ThermoFisher Scientific, MAB5871, 1:5000), anti-phosphoSmad3 (Santa Cruz Biotechnology, sc-517575, 1:500), anti-Smad3 (ThermoFisher Scientific, 51–1500, 1:5000), anti-Flag (Millipore Sigma, F3165, 1:1000) and anti-Glyceraldehyde-3-phosphate dehydrogenase (GAPDH, Abcam, ab9485 1:4000). After incubation with the primary antibodies, membranes were washed for 30 min in TBS-T and incubated with horseradish peroxidase (HRP)-conjugated secondary antibodies: anti-mouse (Santa Cruz Biotechnology, sc-516102, 1:2000), anti-rabbit (Santa Cruz Biotechnology, sc-2357, 1:2000), anti-goat (Millipore Sigma, A5420, 1:5000) or anti-rat (Jackson Immunoresearch, No. 112–035-003, 1:5000) for 90 min at room temperature. The membranes were washed for 60 min and exposed with enhanced chemiluminescence solution (ECL, BioRad) using ImageQuant LAS 4000. The bands were subjected to densitometric analysis using the ImageJ software.

### Immunofluorescence, Hematoxylin and Eosin (H&E), and Von Kossa staining

Kidneys were fixed in 10% formaldehyde, dehydrated, and embedded in paraffin. Tissue sections (5 µm) were prepared and deparaffinized, hydrated, and subjected to antigen retrieval in citrate solution, pH 6, at 95 °C for 30 min. Sections were blocked with 5% donkey serum and labeled with anti-fibronectin (Abcam, ab23750, 1:200) anti-Collagen I (SouthernBiotech, 1310–01, 1:100), anti-αSMA (Millipore Sigma, A2547, 1:200), anti-phosphoMGP (Kind gift of Dr. Schurgers, Maastricht, The Netherlands, 1:100), anti-MGP (Proteintech, 10,734–1-AP, 1:100, for human studies), anti-MGP [59] (1:100, for mouse studies) or anti-phosphoSmad3 (Santa Cruz Biotechnology, sc-517575, 1:200). The slides were subsequently exposed to anti-rabbit AF647 (Jackson Immunoresearch, 1:200), anti-mouse Cy3 (Jackson Immunoresearch, 1:200) or anti-goat AF488 (Jackson Immunoresearch, 1:200). Fluoroshield with 4′,6-diamidino-2-phenylindole (DAPI, Millipore-Sigma) was used for nuclear staining and mounting. A separate set of sections was stained with hematoxylin and eosin (H&E, Sigma-Aldrich), following the manufacturer's protocol or with Von Kossa stain, following the manufacturer's instructions (Abcam). The slides were imaged using a Zeiss AxioObserver.Z2 upright microscope coupled to a Colibiri.7 LED light source. The images were captured with a 10 × objective.

### Kidney biopsies

The study follows the Declaration of Helsinki. All subject recruitment procedures and informed consent forms, including consent to use renal biopsy samples for research purposes, were approved by the Maisonneuve-Rosemont Hospital Research Ethics Board (#2023–3307) and written informed consent was obtained from each patient. Renal biopsy specimens with an adequate amount of tissue for immunofluorescence evaluation after the completion of diagnosis by the Pathology Department of the Maisonneuve-Rosemont Hospital were included. Deidentified human kidney tissue samples from kidney transplant patients (n = 5 IFTA-I, n = 5 IFTA-III, Table S1) were obtained. Paraffin-embedded tissues were cut into 5-μm sections and processed for immunofluorescence as described above.

### Cell culture

The mouse fibroblast cell line NIH3T3 was obtained from the American Type Culture Collection (CRL-1658, USA). The cells were maintained in a humidified atmosphere, 5% CO_2_ and at 37ºC with Dulbecco’s Modified Eagle’s Medium (DMEM, ThermoFisher Scientific) supplemented with 5% fetal bovine serum (FBS, Gibco/Life Technologies). Cell line authentication was performed using short tandem repeat (STR) profiling system [60]. In addition, cells were confirmed to be free of mycoplasma contamination by a PCR-based assay [61]. Primary fibroblasts were isolated according to a previously reported protocol [62] and maintained in DMEM media supplemented with 20% fetal bovine serum.

### Traction force microscopy (TFM)

To create polyacrylamide hydrogels, we followed standard methods [63–65]. Briefly, we mixed 4 µL of 40% acrylamide (Bio-Rad, 161–0140) and 2% bis-acrylamide (Bio-Rad, 161–0142) in glass-bottom 35 mm dishes (IBIDI, 81,218–200). The dishes were pre-treated with (3-aminopropyl)triethoxysilane (Sigma-Aldrich, 741,442) and 25% glutaraldehyde (Electron Microscopy Sciences, #16,200). We used 55 kPa hydrogels. Polyacrylamide mixing ratios are available in previously published resources [64]. We covered the gel with a 12 mm poly-l-lysine-coated cover glass (Sigma 25,988–63-0) containing dark red fluorescent microbeads (ThermoFisher, F8807) on the surface, then removed the coverslip, thereby transferring beads to the TFM gel. To prepare for cell seeding, we activated the gels using sulfo-SANPAH (ThermoFisher, 22,589) and coated them with fibronectin (Millipore Sigma FC010). To measure cellular force, two images were needed: one with no cells and another with adhered cells exerting traction. Cells were seeded on TFM hydrogel 12 h before traction experiments. Imaging was performed in a temperature-controlled incubator (IBIDI, 10,720). We conducted a 45-min timelapse imaging session, capturing images every 3 min in 60 different microscope stage positions. To acquire the reference frame, we added 2 mL of 2% Triton-X-100 (Millipore Sigma, 9036–19-5) in ddH2O at 37 °C to the 1 mL of culture medium to detach cells. For imaging, we used a Nikon Eclipse Ti2 equipped with a 20X 0.75 NA objective and a 1.5X magnification lens. The resulting pixel size on the image is 0.37 microns. Images of cells were obtained in bright-field mode and those of beads with epi fluorescence. For image analysis, we trained a custom model for each cell type by fine-tuning the “cyto” model from Cellpose V2 with 3–10 images of 10–20 cells [66]. We tracked individual cells using a nearest neighbor algorithm based on the centroids of their masks [67]. Bead displacements were measured using MATLAB functions: imregDemons and vision.PointTracker. Force fields were computed using Fourier Transform Traction Cytometry. Cellular mechanical work was calculated as the average of instantaneous work over the entire time-lapse.

### Statistical analysis

The results are presented as means ± standard error. For multiple group comparisons, the statistical significance was calculated by one-way analysis of variance (ANOVA) followed by Dunnett’s or Šídák post-hoc test. To compare between two groups, an unpaired, two-tailed Student’s t-test was performed. We considered *p-values* < 0.05 as statistically significant. Statistical analysis and graphical representation were performed using GraphPad Prism v9.1.0. Additional details for methods and materials are provided in the supplementary information file.

## Supplementary Information

Supplementary Material 1.

## Data Availability

All data are available in the main text or the supplementary materials.
