# Supplementary material for: Matrix Gla Protein is a novel regulator of TGFβ–dependent fibroblast activation and kidney fibrosis
Source: Mol Biomed. 2026 Aug 6;7:127. doi: 10.1186/s43556-026-00518-0 (PMC13442784; doi:10.1186/s43556-026-00518-0)
Supplement: Supplementary file 1 [file 43556_2026_518_MOESM1_ESM.pdf]

Supplementary Information For: Matrix Gla Protein is a novel regulator of TGFβ–dependent fibroblast activation and kidney fibrosis

Jonatan Barrera-Chimal[1,#], Jocelyn López-Ramírez[1,2,#], Nathalie Henley[1], Peng Gao[1], Yeye Ma[1,2], Nicolas Desjardins-Lecavalier[1], Santiago Costantino[1,3], Virginie Royal[1], Stijn M. Agten[4], Teresa Borrás[5], Vincent Pichette[1], Darren A. Yuen[6], Leon J. Schurgers[4] and Casimiro Gerarduzzi[1,2]*

[1]Centre de Recherche de l'Hôpital Maisonneuve-Rosemont, Centre affilié à l'Université de Montréal, Montréal, Québec, Canada.

[2]Département de Médecine, Faculté de Médecine, Université de Montréal, Montréal, Québec, Canada.

[3]Département d'Ophtalmologie, Faculté de Médecine, Université de Montréal, Montréal, Québec, Canada.

[4]Department of Biochemistry, Cardiovascular Research Institute Maastricht (CARIM), Maastricht University, Maastricht, The Netherlands.

[5]Department of Ophthalmology, University of North Carolina School of Medicine, Chapel Hill, NC, USA.

[6]Keenan Research Centre for Biomedical Science, Li Ka Shing Knowledge Institute, St. Michael's Hospital (Unity Health Toronto) and Department of Medicine, University of Toronto, Toronto, Ontario, Canada.

[#]Contributed equally

***Corresponding author:** Dr. Casimiro Gerarduzzi
casimiro.gerarduzzi@umontreal.ca

Supplementary Materials and Methods

Mgp transfection and MGP recombinant protein treatment

For *Mgp* overexpression studies, the cells were grown to 70-80% confluence and transfected with 2 µg of Myc-*MGP* (Origene, No. RC210666), Flag-*BMP-2* (SinoBiological Inc, No. HG10426-NF), Myc-empty vector or the different mutant versions of the Myc-*MGP* vector using JetPrime transfection reagent (Polyplus, VWR Canada) in a 6-well dish following the manufacturer's protocol, and the cells were harvested after 24 hours. For the silencing studies, cells were seeded in a 6-well dish at 70-80% confluence and were transfected with an *Mgp* mouse shRNA Plasmid (Origene, No. TL501343) or control shRNA using JetPrime transfection reagent (Polyplus; VWR, Canada), following the manufacturer's protocol for 24 h, followed by a 5 ng/mL treatment with recombinant TGFβ (PeproTech) for an additional 24 h. For the recombinant protein treatments, the cells were plated overnight in a 6-well dish at a 60% confluence, starved with 1% FBS-DMEM for 4 h and then treated for 24 h with 50 ng/mL of recombinant MGP protein (Novus Biologicals, No. NBP1-72441), 5 ng/mL treatment with TGFβ (PeproTech), 50 ng/mL of recombinant BMP-2 (Fisher Scientific), and a combination of MGP, TGFβ, and/or BMP-2 according to the indicated concentrations or PBS vehicle diluted in fresh media. The Smad3 inhibitor, SIS3 (MedChemExpress) was used at 1µM.

MGP mutagenesis

To generate *MGP* serine (S22, S25, S28 and S3X) and glutamic acid (E21, E56, E60, E67, E71, E3X) mutants, we performed site-directed mutagenesis using the Q5® Site-Directed Mutagenesis Kit (New England Biolabs) according to the manufacturer's instructions and generation of mutant sites was verified by Sanger sequencing at the genomic platform of the Institut de Recherche en Immunologie et Cancérologie, Université de Montréal.

Co-Immunoprecipitation

NIH3T3 cells were transfected in 10-cm dishes with empty vector, *MGP*, or *BMP-2*, respectively. Medium was collected 48 h after transfection and centrifuged at 5,000 g for 10 min. The supernatant was then filtered through a 0.22 µm Millex-GV PVDF membrane filter. Prior to the addition of Myc-agarose beads (Millipore Sigma, A7470), the medium from MGP-overexpressing cells was combined with medium from cells transfected with empty vector or BMP-2 and incubated for 30 min at room temperature. Myc-beads were added to the medium and incubated overnight.

The next day, samples were centrifuged once at 8,000 rpm for 3 min to recover the supernatant, followed by four washes at 8,000 rpm for 3 min each with low-salt wash buffer. For elution, 40 µL of Laemmli buffer was added to the beads, and then heated at 95 °C for 10 min. The samples were analyzed by western blotting as described in the main text of the manuscript.

Scratch assay

NIH3T3 cells were seeded in triplicate on a 96-well plate and allowed to adhere overnight. Next, transfections or treatments with recombinant proteins were performed as described in the main text. A wound maker (Sartorius Canada, ON) was used to create identical scratches in the monolayer of cells. The plate was photographed every 4 hours for 48 h to monitor the wound closure dynamics using the Incucyte Live Cell SX5 system (Sartorius). The data were analyzed with the Incucyte software.

Phalloidin staining

Cytoskeletal F-actin was visualized using Tetramethylrhodamine isothiocyanate (TRITC)–conjugated Rhodamine Phalloidin (Thermo Fisher Scientific) at 1:500 in PBS for 1 hour. 4,6-Diamidino-2-phenylindole (Sigma-Aldrich) was used for nuclear staining (blue). The slides were imaged using a Zeiss AxioObserver.Z2 upright microscope coupled to a Colibiri.7 LED light source.

RNA sequencing analyses

RNA sequencing data were downloaded from the GEO dataset GSE135327[25], comprising 18 transplant kidney biopsies (disease group, 9 had minimal-mild interstitial fibrosis IFTA; 0–1 and 9 had moderate-severe fibrosis; IFTA 2–3) and 12 normal control samples. Expression values were log2-transformed after adding a pseudocount of 1. Lowly expressed genes were filtered out (expression > 1 in ≥20% of samples). *MGP* expression levels were compared between disease and normal groups using Wilcoxon rank-sum test. For downstream analyses, disease samples were divided into *MGP*-high and *MGP*-low groups based on median *MGP* expression. A sensitivity analysis was performed using tertile-based grouping. Differentially expressed genes between *MGP*-high and *MGP*-low groups were identified using limma with thresholds of |logFC| > 1 and false discovery rate (FDR) < 0.05. GSEA was performed using the clusterProfiler R package v4.16.0 with gene sets from Hallmark collection Molecular Signatures Database (MSigDB) and

Reactome pathway database and the enrichment significance was set at FDR < 0.25 for visualization. Furthermore, a fibroblast–TGFβ–regulated gene signature, consisting of 54 genes[25], was used for single-sample GSEA analysis (ssGSEA). The association between *MGP* expression levels and fibroblast–TGFβ signature genes was assessed using Pearson correlation.

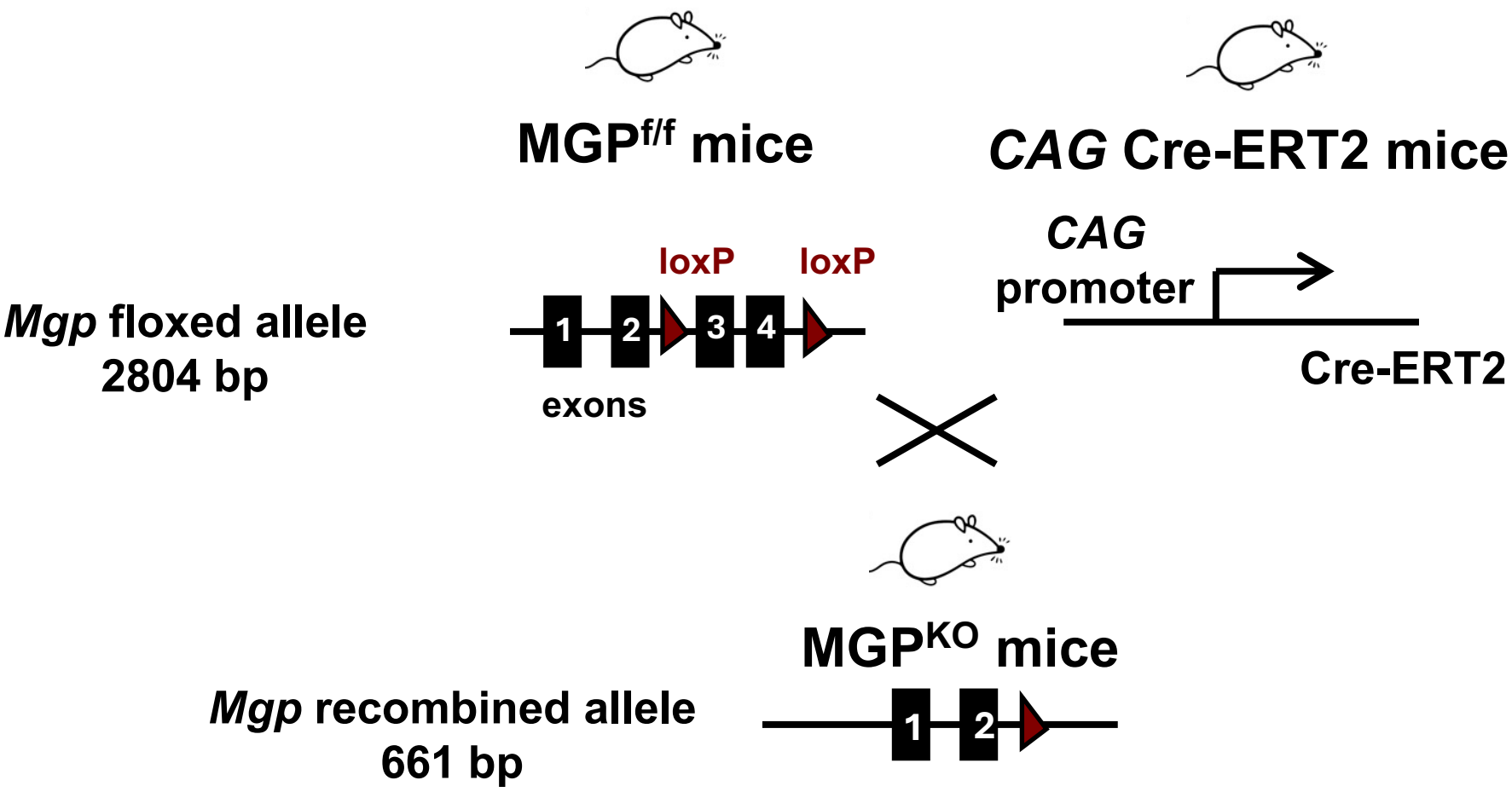

Sup Fig 1. Mice model of ubiquitous and inducible *Mgp* knockout. Schematic representation of the breeding strategy between *Mgp* floxed (MGP$^{f/f}$) mice and mice expressing the Cre-ERT2 recombinase under the control of the chicken β-actin promoter/enhancer coupled to the cytomegalovirus (CMV) immediate-early enhancer (CAG-Cre-ERT2), generating mice with ubiquitous and inducible *Mgp* knockout (MGP^{KO}) following tamoxifen administration.

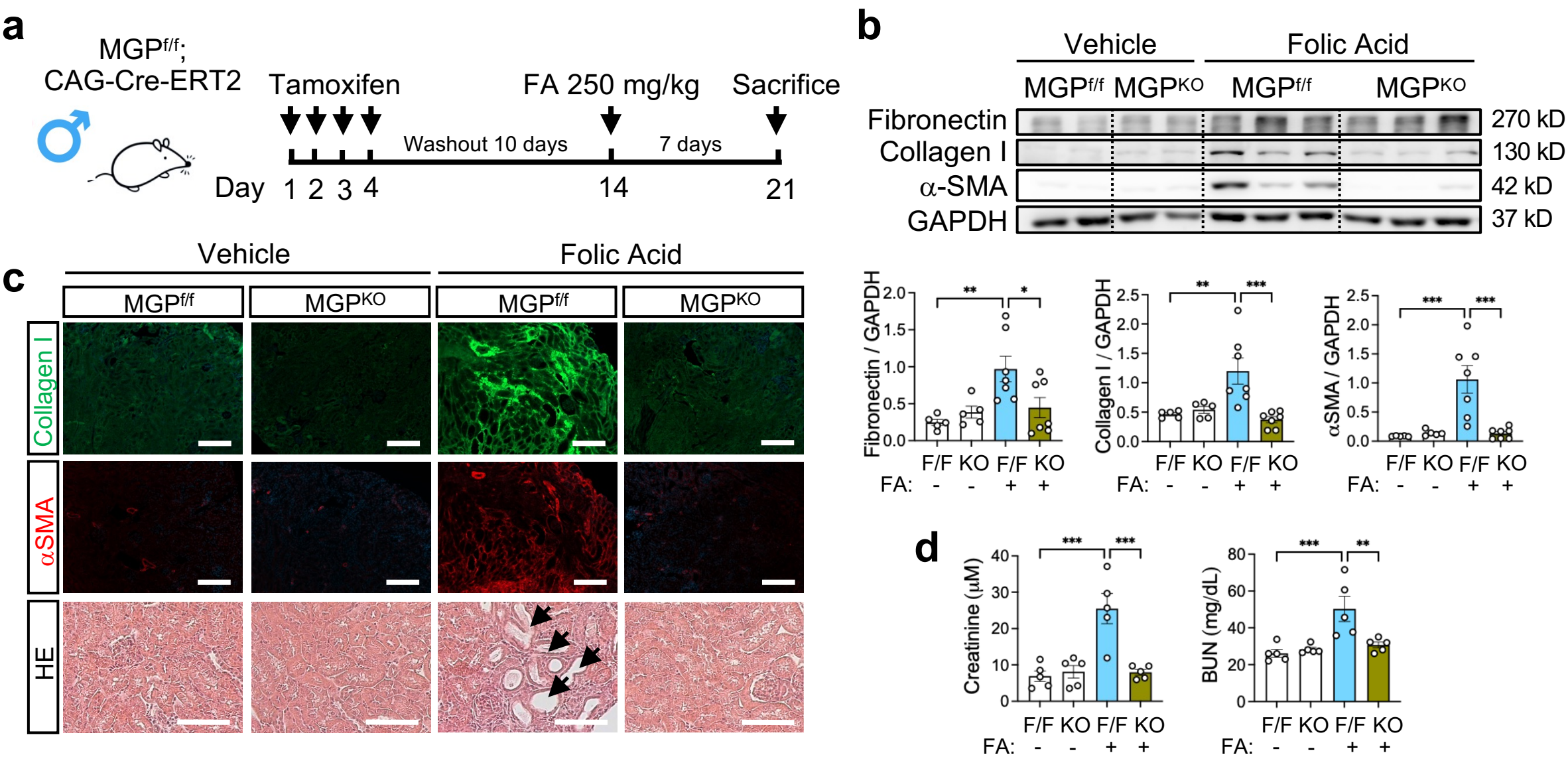

Sup Fig 2. Inducible and ubiquitous *Mgp* knockout protects against folic acid induced kidney fibrosis in male mice. a) Experimental design; male mice were injected with tamoxifen for four consecutive days, and after a 10-day washout period, they received a single folic acid injection (250 mg/kg). The mice were sacrificed at day 7 post-FA injection. b) Western blot analysis was performed to evaluate the kidney levels of fibronectin, collagen I, αSMA (α-smooth muscle actin), and GAPDH. Upper insets are representative blots, and the graphs depict the densitometric analyses (Veh $MGP^{F/F}$ n=5, Veh MGP^{KO} n=5, FA $MGP^{F/F}$ n=7, FA MGP^{KO} n=7). c) Immunofluorescence images of kidneys from $MGP^{f/f}$ or MGP^{KO} mice following vehicle or FA injections showing collagen I (green) or αSMA (red) and representative images of H&E staining (revealing tubular injury). Images are representative of n = 5 mice per group. Scale bar: 100 μm. d) Plasma creatinine and urea were determined (Veh $MGP^{F/F}$ n=5, Veh MGP^{KO} n=5, FA $MGP^{F/F}$ n=5, FA MGP^{KO} n=5). Data are presented as mean ± SEM and were analyzed using a one-way ANOVA followed by Šídák post-hoc test (b, d). *p<0.05, **p<0.01 and ***p<0.001.

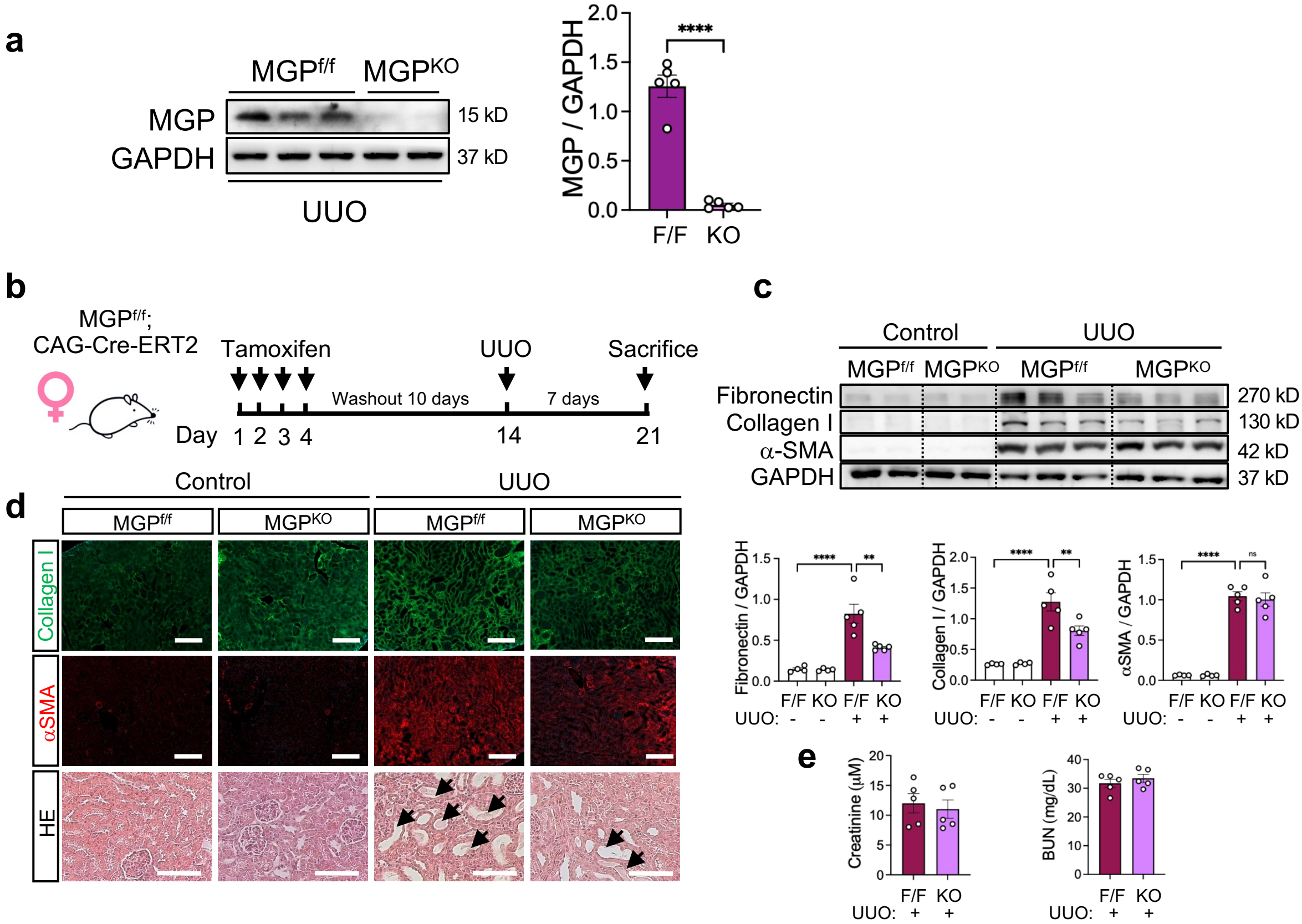

Sup Fig 3. Ubiquitous *Mgp* knockout protects against UUO induced kidney fibrosis. a) Western blot analysis was performed in kidney tissue lysates from mice underwent UUO to induce kidney fibrosis and to evaluate the kidney levels of MGP and GAPDH. b) Experimental design; female mice were injected with tamoxifen for four consecutive days and after a 10-day washout period underwent UUO surgery UUO. The mice were sacrificed at day 7 post-UUO. c) Western blot analysis showing the levels of fibronectin, collagen I, αSMA (α-smooth muscle actin), and GAPDH, upper insets are representative blots, and the graphs depict the densitometric analyses (CTL MGP$^{F/F}$ n=4, CTL MGP^{KO} n=4, UUO MGP$^{F/F}$ n=5, FA MGP^{KO} n=5). d) Immunofluorescence images of kidneys from MGP$^{f/f}$ or MGP^{KO} mice following control or UUO surgery showing collagen I (green) or αSMA (red) and representative images of H&E staining. e) Plasma creatinine and urea were determined (UUO MGP$^{F/F}$ n=5, FA MGP^{KO} n=5). Data are presented as mean ± SEM and were analyzed using Student's t-test (a) or a one-way ANOVA followed by Šídák post-hoc test (c, e). *p<0.05, **p<0.01, ***p<0.001 and ****p<0.0001. Images are representative of n = 5 mice per group (d). Scale bar: 100 μm.

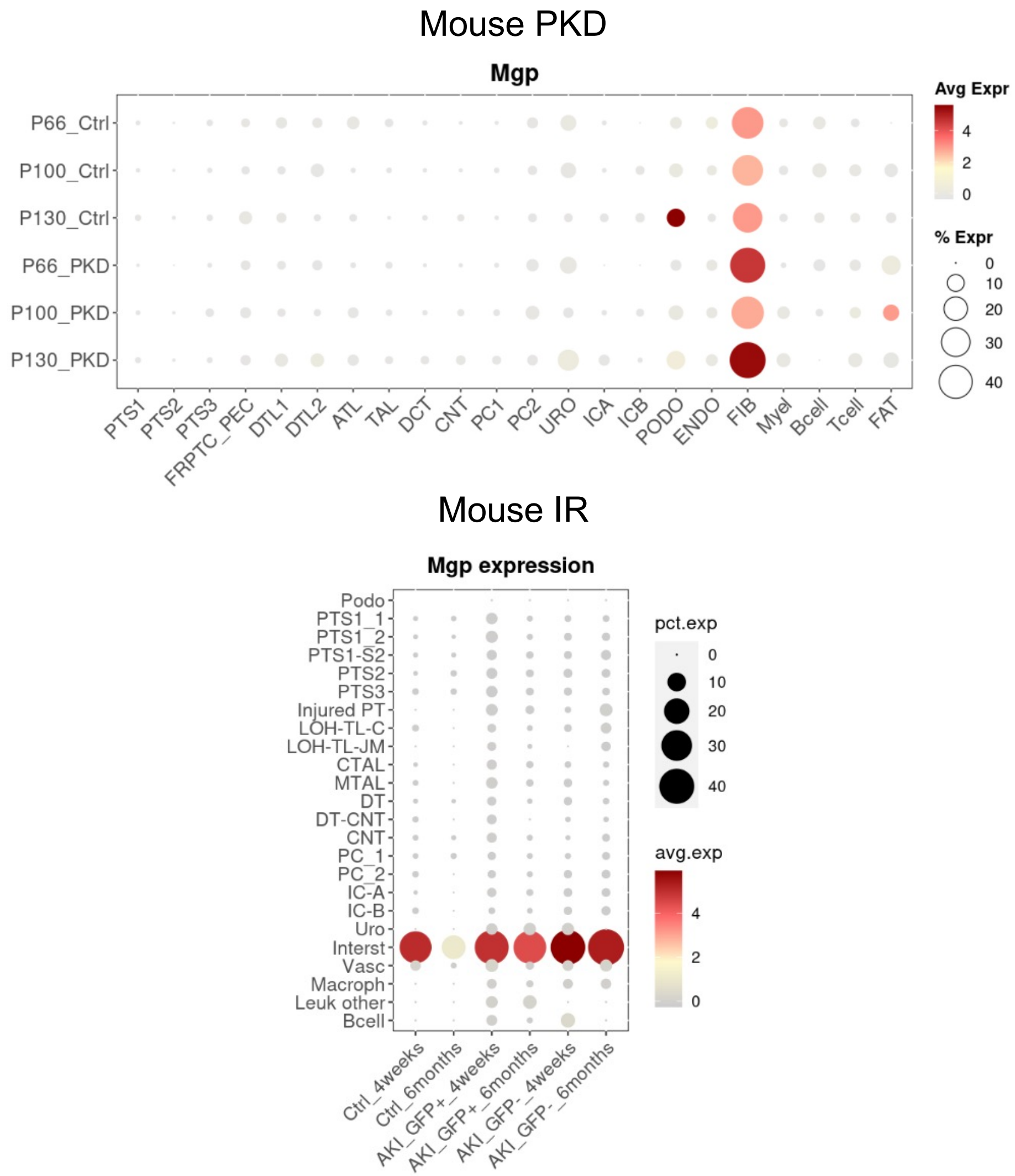

Sup Fig 4. *Mgp* expression is specifically induced in fibroblasts. *Mgp* mRNA levels in different cell subsets as detected by single-nucleus RNA sequencing in the mouse model of Polycystic kidney disease (PKD) and ischemia/reperfusion injury (IR). The data was generated using the http://humphreyslab.com/SingleCell/ website.

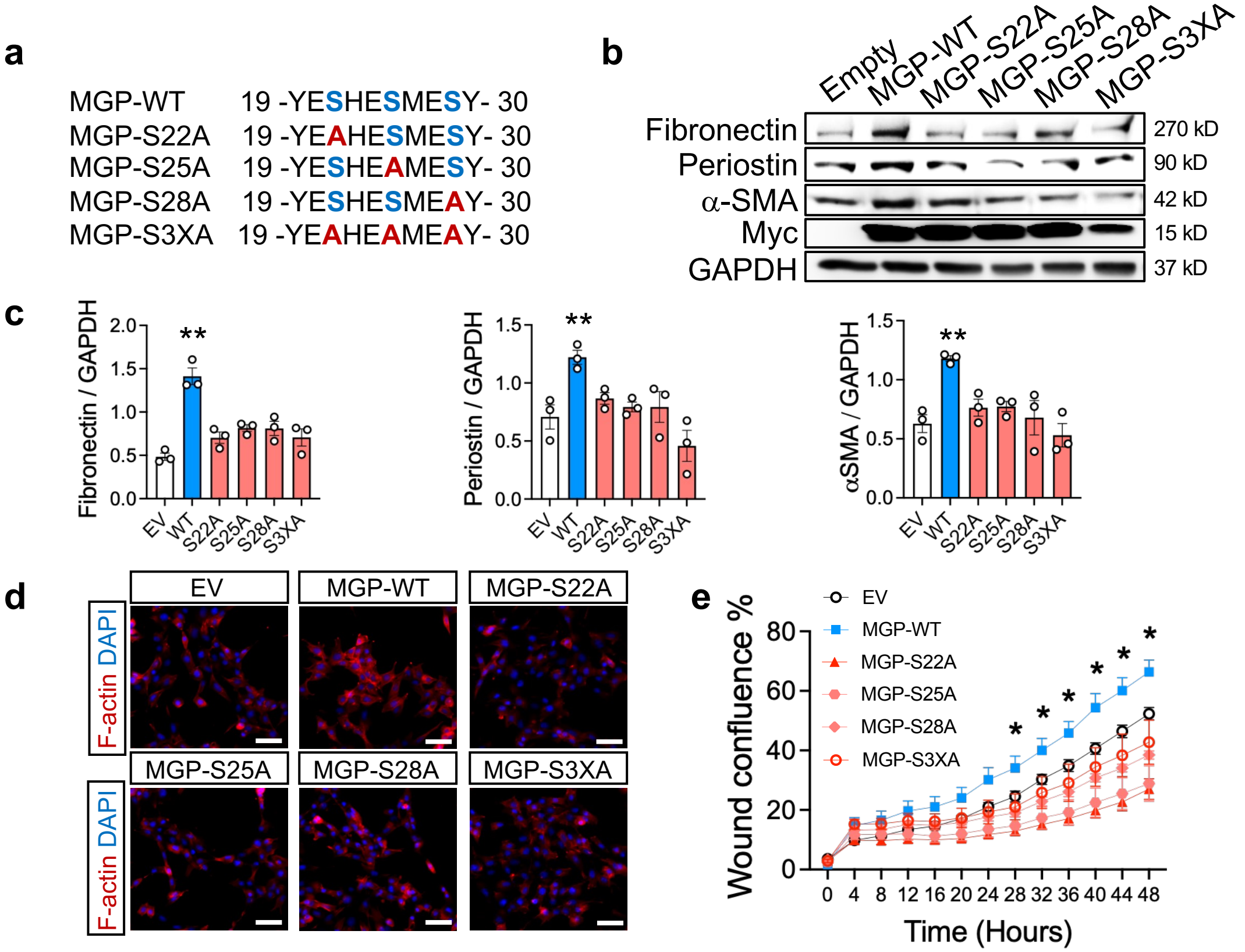

Sup Fig 5. MGP phosphorylation is required for MGP-induced FMT. a) Site-directed mutagenesis was performed to substitute the serine residues, in which MGP is phosphorylated (S22, S25 and S28), to alanine. The amino acid sequence of the mutated region is shown for the wild-type (MGP-WT) and for the mutated MGP versions, MGP-S22A, MGP-S25A, MGP-S28A and MGP-S3XA. b) NIH3T3 cells were transfected with the WT and mutant versions of MGP and 24 hours after the cell lysates were analyzed by western blot to evaluate the levels of fibronectin, periostin, αSMA (α-smooth muscle actin), Myc and GAPDH (n = 3 per group). c) Densitometric analysis are shown (n = 3 per group). d) Phalloidin-Rhodamine staining was performed 24 hours after MGP-WT or MGP-mutants transfection to assess for stress fiber formation. Scale bar: 10 µm. Images are representative of three independent replicates. e) NIH3T3 cells were plated to form a monolayer, and a scratch-wound was performed to evaluate the migration capacity of transfected cells with the different MGP WT or mutant versions. The wound confluence % that was monitored every 4 hours (n = 5 per group). Data are presented as mean ± SEM and were analyzed using a one-way ANOVA followed by Šídák post-hoc test (c, e). *$p<0.05$ and **$p<0.01$ vs. EV.

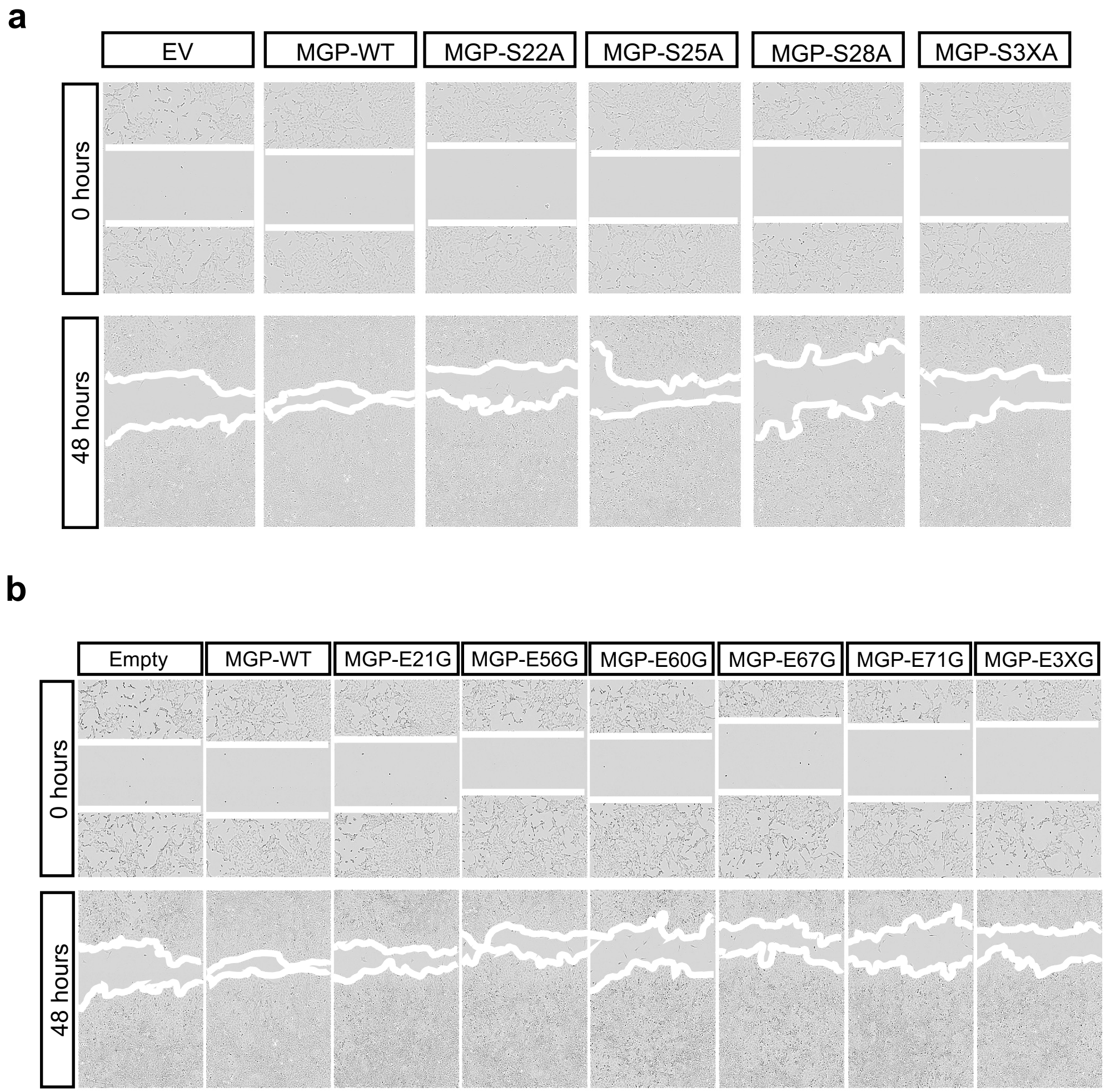

Sup Fig 6. Effect of serine to alanine substitutions and glutamic acid to glycine substitutions of MGP on migration capacity of fibroblasts. a) Representative images of five independent replicates from the scratch-wound assay are shown for the cells transfected with empty vector (EV), wild-type MGP or the different serine mutant MGP versions at baseline (0 hours) and 48 hours. b) Representative images of five independent replicates from the scratch-wound assay are shown for the cells transfected with empty vector (EV), wild-type MGP or the different glutamic acid mutant MGP versions at baseline (0 hours) and 48 hours.

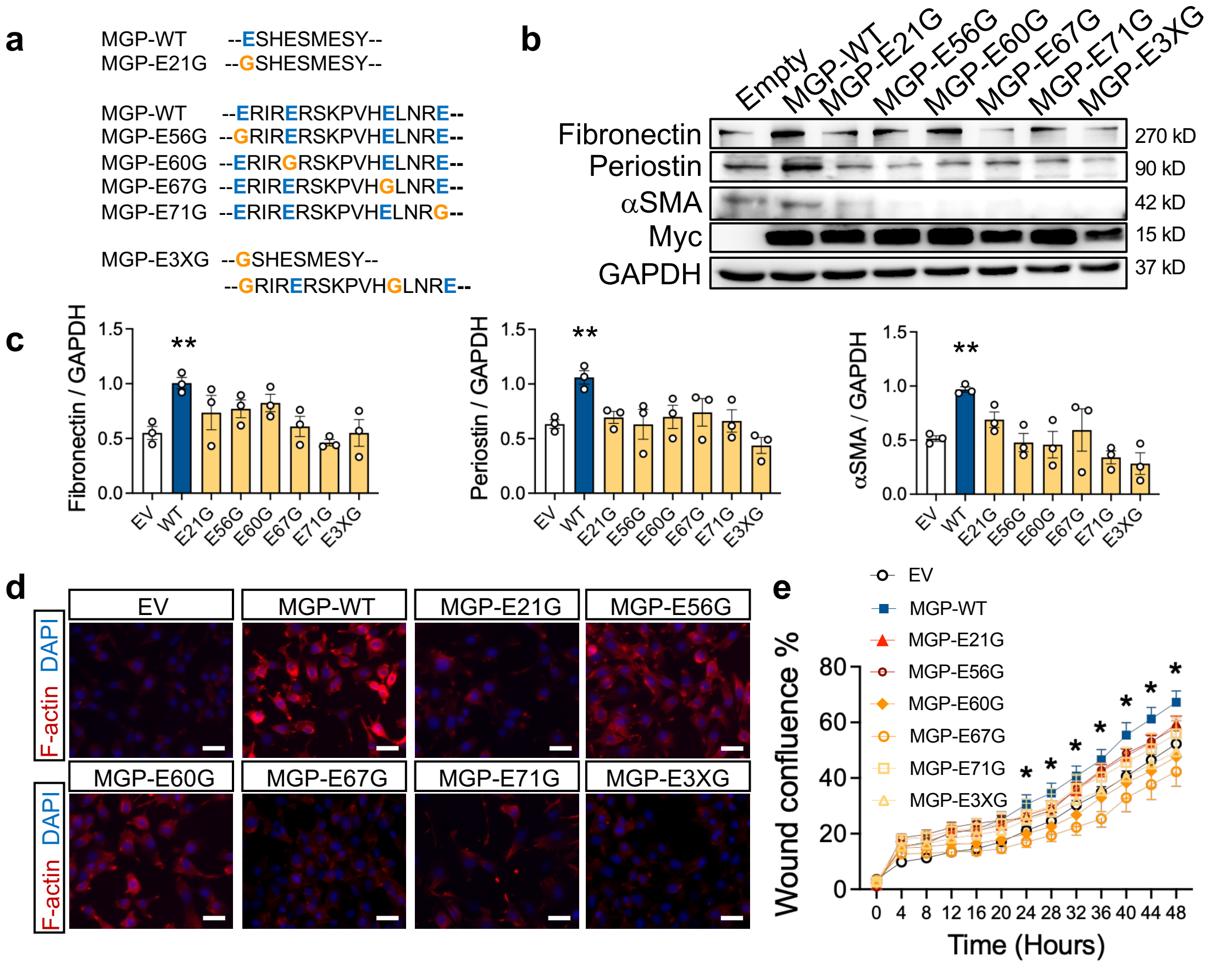

Sup Fig 7. MGP carboxylation is required for MGP-induced FMT. a) Site-directed mutagenesis was performed to substitute the glutamic acid residues, in which MGP is carboxylated (E21, E56, E60, E67 and E71), to glycine. The amino acid sequence of the mutated region is shown for the wild-type (MGP-WT) and for the mutated MGP versions, MGP-E21G, MGP-E56G, MGP-E60G, MGP-E67G, MGP-E71G and MGP-E3XG. b) NIH3T3 cells were transfected with the WT and mutant versions of MGP, and 24 hours after, the cell lysates were analyzed by western blot to evaluate the levels of fibronectin, periostin, αSMA (α-smooth muscle actin), Myc and GAPDH (n = 3 per group). c) Densitometric analysis are shown (n = 3 per group). d) Phalloidin-Rhodamine staining was performed 24 hours after MGP-WT or MGP-mutants transfection to assess for stress fiber formation. Scale bar: 10 µm. Images are representative of three independent replicates. e) Scratch-wound assay to evaluate the migration capacity of transfected NIH3T3 cells with the different MGP WT or mutant versions. The wound confluence % that was monitored every 4 hours (n = 5 per group). Data are presented as mean ± SEM and were analyzed using a one-way ANOVA followed by Šídák post-hoc test (c, e). *$p<0.05$ and **$p<0.01$ vs. EV.

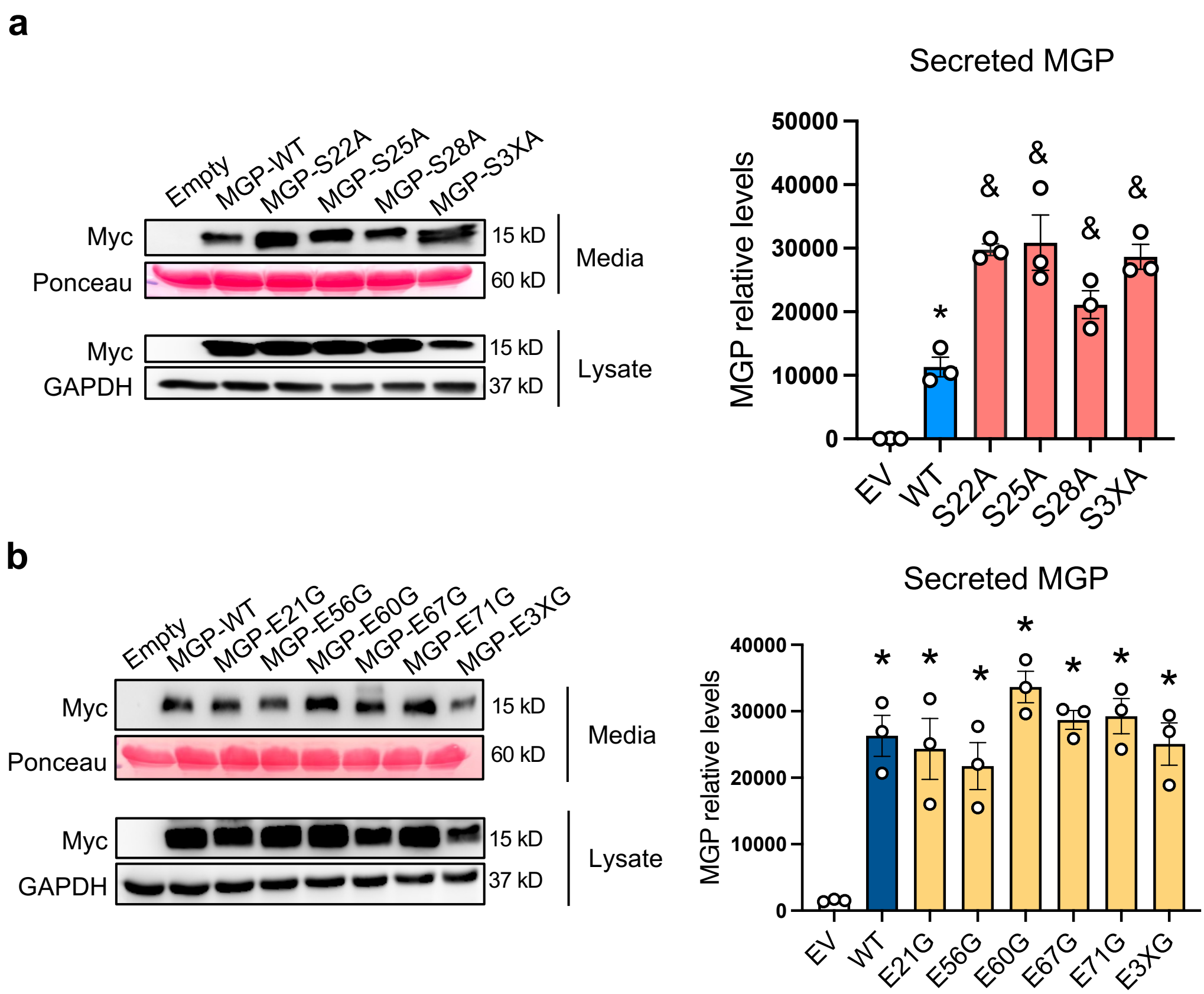

Sup Fig 8. Effect of serine to alanine substitutions and glutamic acid to glycine substitutions on MGP secretion. NIH3T3 cells were transfected with empty vector (EV), wild-type *MGP*-myc or the different serine (a) and glutamic acid (b) mutant versions of *MGP*. The medium of the cells was collected and the amount of secreted MGP in the medium was assessed by western blot through Myc detection. Ponceau staining was used as a loading control. The western blot images are representative from the results obtained in the medium and cell lysates. The graph represents the densitometric analysis of the secreted MGP (n = 3 per group). Data are presented as mean ± SEM and were analyzed using a one-way ANOVA followed by Šídák post-hoc test. *p<0.01 vs EV, & p<0.01 vs MGP-WT.

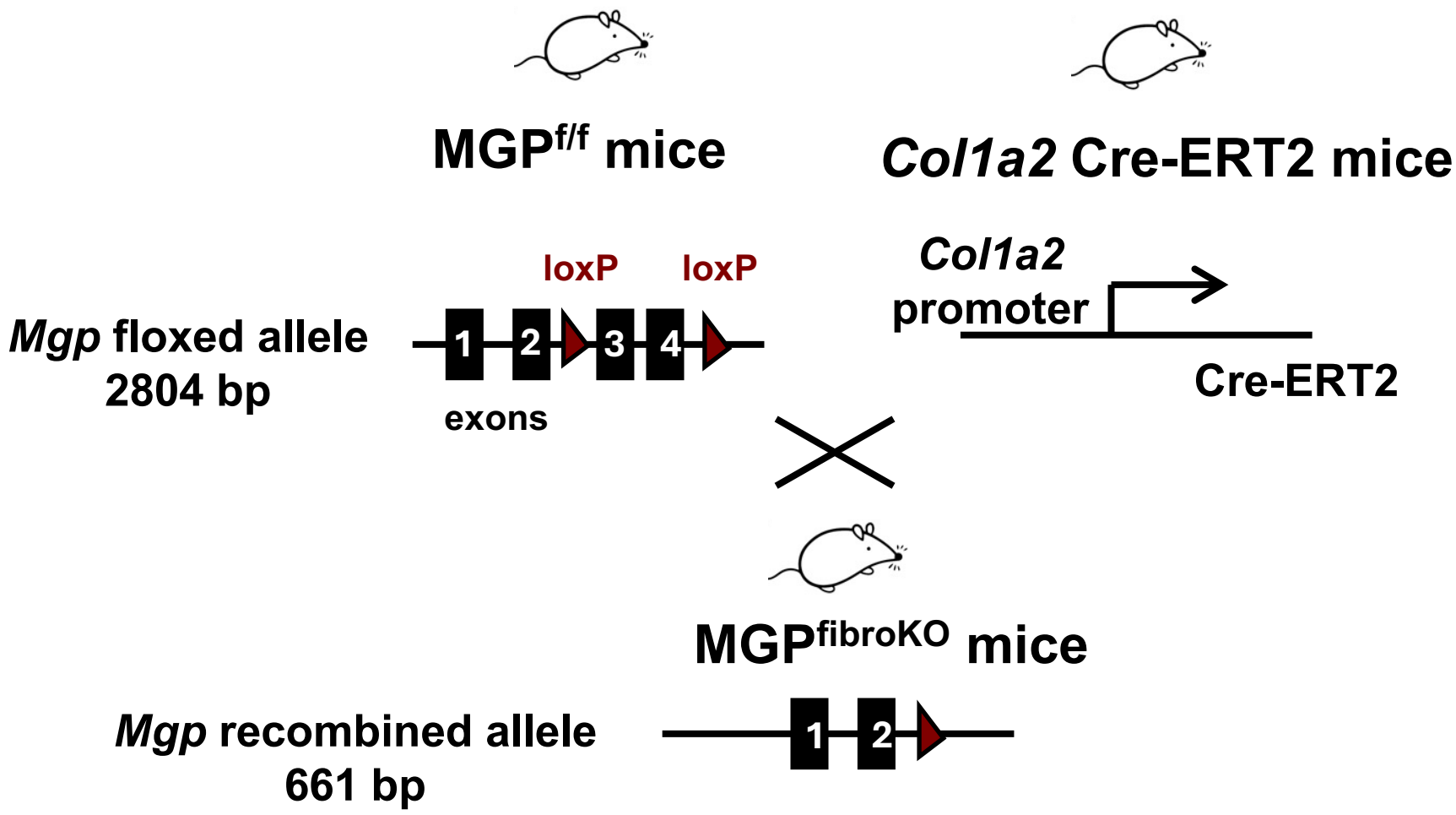

Sup Fig 9. Mice model of inducible and fibroblast-specific *Mgp* knockout. Schematic representation of the breeding strategy between *Mgp* floxed ($MGP^{f/f}$) mice and mice expressing the Cre-ET2 recombinase under the control of the *Colagen1a2* promoter (Col1a2-Cre-ET2), generating mice with inducible and fibroblast specific MGP knockout ($MGP^{fibroKO}$) following tamoxifen administration.

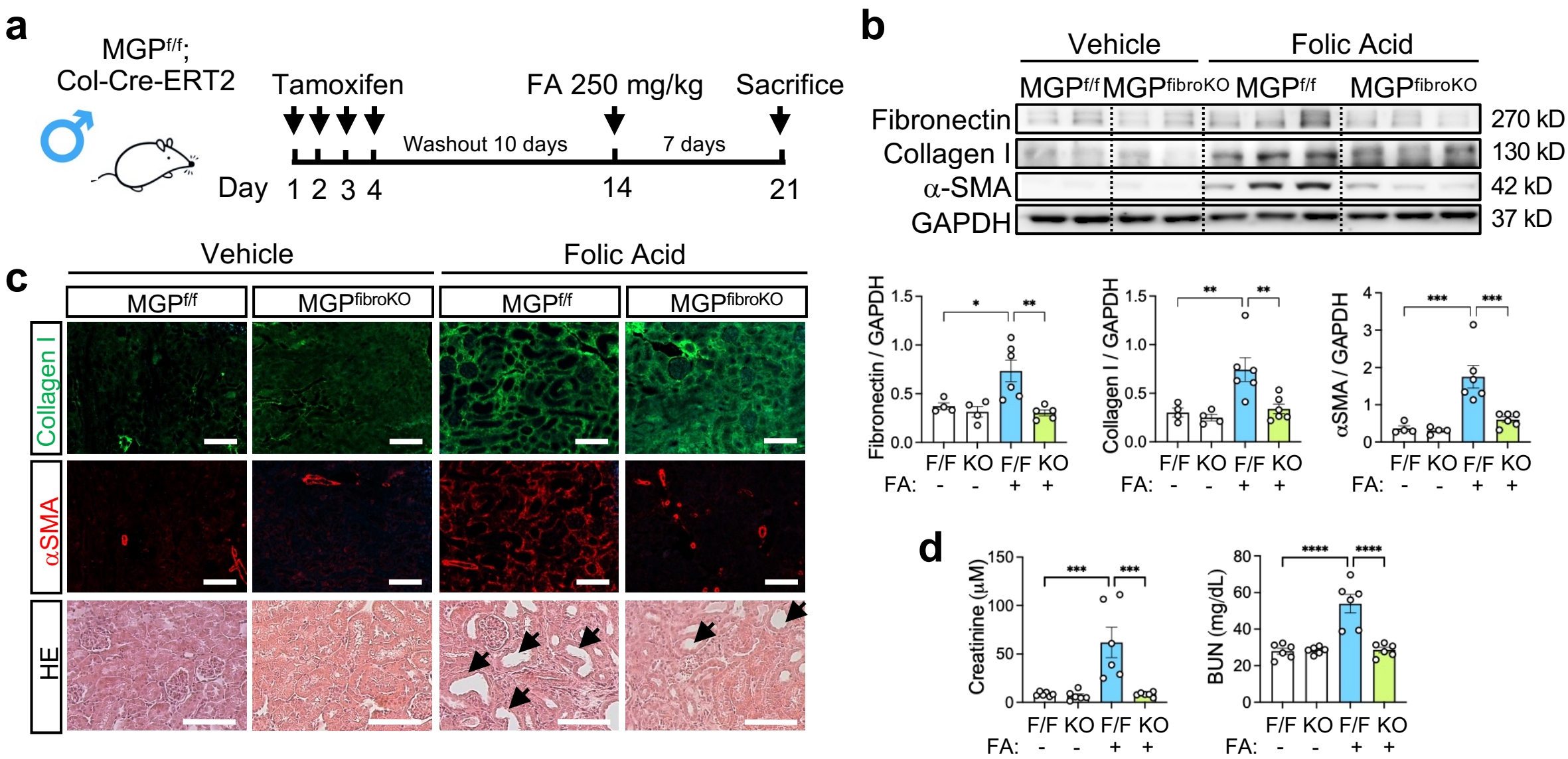

Sup Fig 10. Fibroblast-specific *Mgp* knockout protects against FA-induced kidney fibrosis in male mice. a) Experimental design; male mice were injected with tamoxifen for four consecutive days, and after a 10-day washout period, they received a single folic acid injection (250 mg/kg). The mice were sacrificed at day 7 post-FA injection. b) Western blot analysis was performed to evaluate the kidney levels of fibronectin, collagen I, αSMA (α-smooth muscle actin), and GAPDH. Upper insets are representative blots, and the graphs depict the densitometric analyses (Veh $MGP^{F/F}$ n=4, Veh $MGP^{fibroKO}$ n=4, FA $MGP^{F/F}$ n=6, FA $MGP^{fibroKO}$ n=6). c) Immunofluorescence images of kidneys from $MGP^{f/f}$ or $MGP^{fibroKO}$ mice following vehicle or FA injections showing collagen I (green) or aSMA (red) and representative images of H&E staining (revealing tubular injury). Images are representative of n = 5 mice per group. Scale bar: 100 µm. d) Plasma creatinine and urea were determined (Veh $MGP^{F/F}$ n=6, Veh $MGP^{fibroKO}$ n=6, FA $MGP^{F/F}$ n=6, FA $MGP^{fibroKO}$ n=6). Data are presented as mean ± SEM and were analyzed using a one-way ANOVA followed by Šídák post-hoc test (b, d). *p<0.05, **p<0.01, ***p<0.001 and ****p<0.0001.

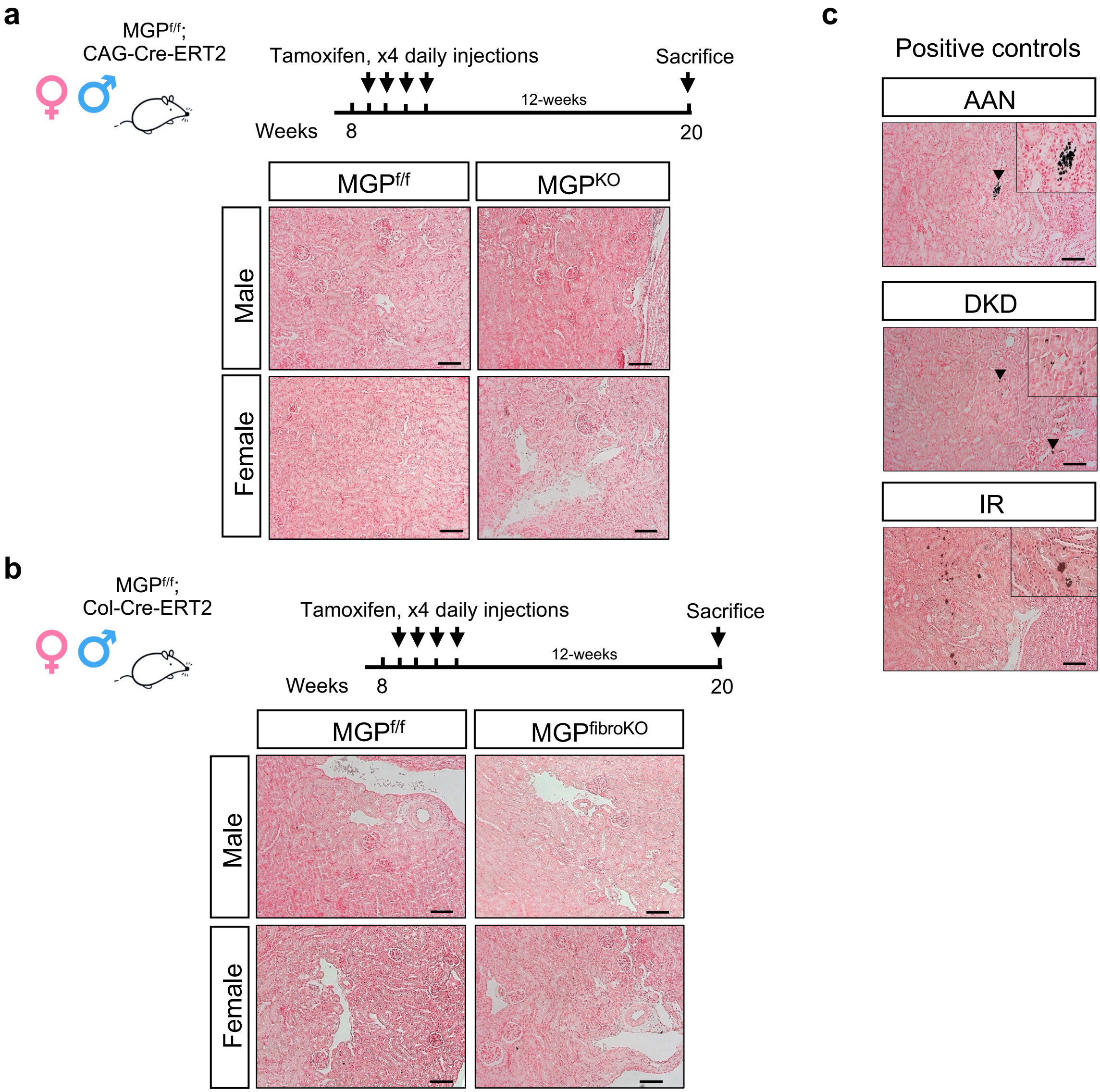

Sup Fig 11. Inducible whole body or fibroblast-specific *Mgp* knockout does not associate with calcification in the kidney. Representative Von Kossa staining is shown for kidney tissues from mice treated with tamoxifen at 8 weeks of age to induce MGP recombination and followed up for 12 additional weeks to evaluate vascular calcification in a) whole-body MGP KO mice (MGP^{KO}) and b) fibroblast-specific KO mice ($MGP^{fibroKO}$) as compared to control littermates ($MGP^{f/f}$). c) As positive controls for calcification, kidney samples from mice under aristolochic acid nephropathy (AAN) treatment, diabetic kidney disease (DKD) or ischemia/reperfusion injury (IR) were used. Images are representative of n = 5 mice per group. Scale bar: 100 µm

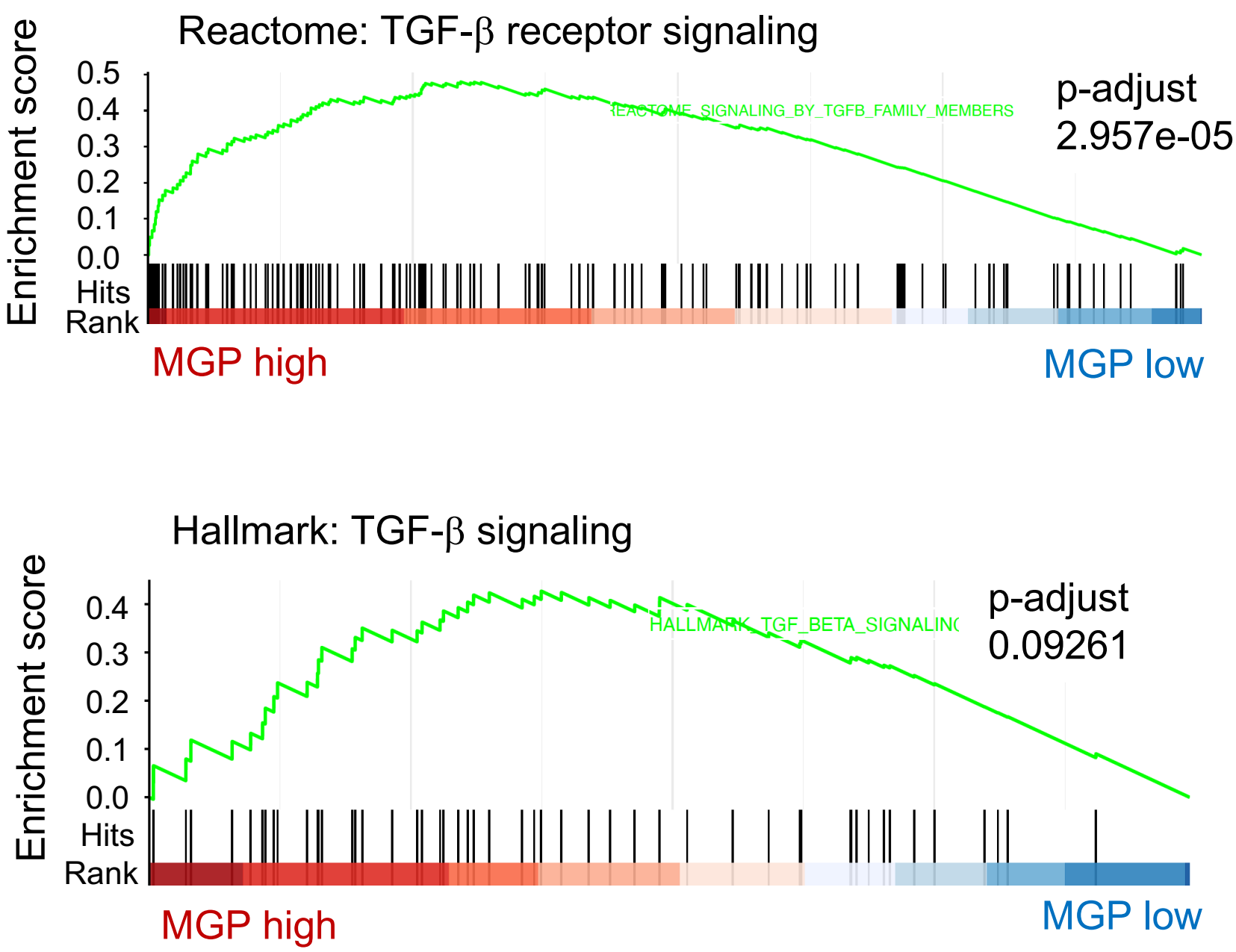

Sup Fig 12. *MGP* high samples show enriched TGFβ signaling. Gene set enrichment analysis (GSEA) of TGFβ–related pathways in *MGP*-high versus *MGP*-low disease samples.

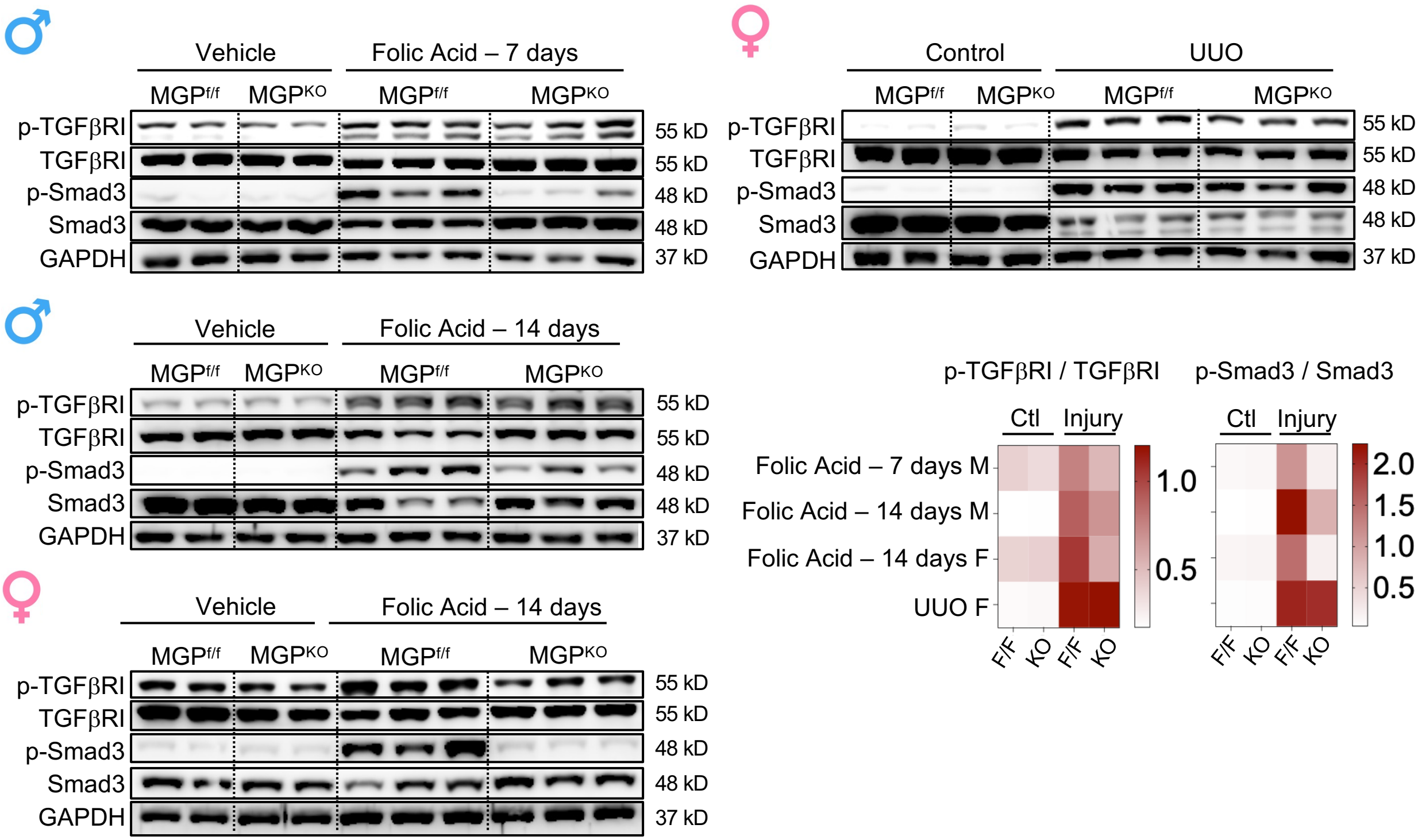

Sup Fig 13. Ubiquitous *Mgp* deficiency inhibits TGFβ signaling in FA and UUO induced kidney fibrosis. Western blot analysis was performed in kidney tissue lysates from the different cohorts of male and female mice that received FA injection or underwent UUO surgery to evaluate the kidney levels of phospho-TGFβRI (TGFβ receptor I), TGFβRI, phospho-Smad3, Smad3 and GAPDH. Representative blot images for each of the mice cohorts are shown, and the heatmaps represent the densitometric analysis (For FA cohorts: Veh $MGP^{F/F}$ n=4, Veh $MGP^{fibroKO}$ n=4, FA $MGP^{F/F}$ n=5, FA $MGP^{fibroKO}$ n=5 / For UUO cohort: CTL $MGP^{F/F}$ n=4, CTL $MGP^{fibroKO}$ n=4, UUO $MGP^{F/F}$ n=5, UUO $MGP^{fibroKO}$ n=5).

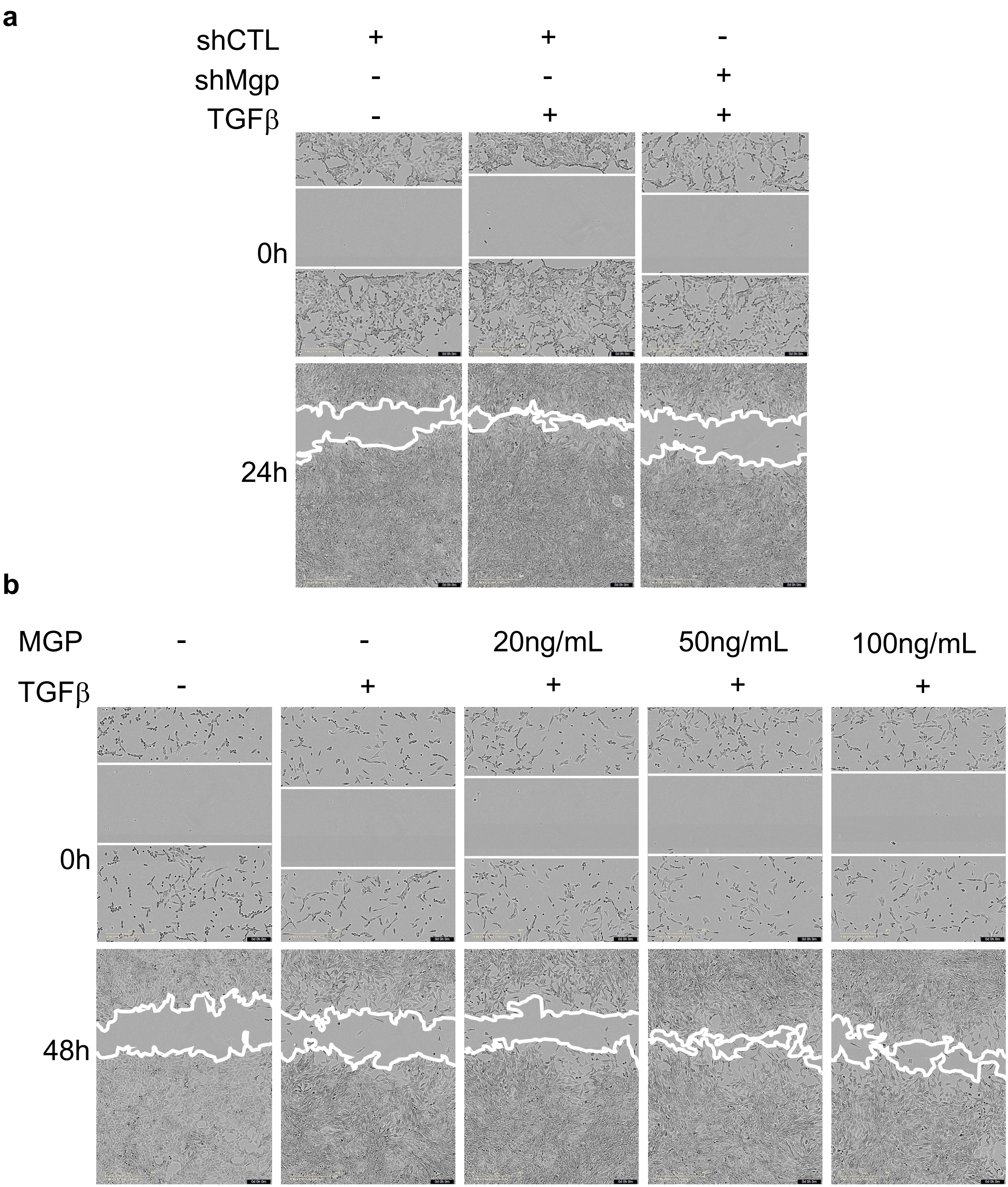

Sup Fig 14. Effect of MGP on the TGFβ induced migration capacity of fibroblasts. **a**) Representative images of three independent replicates from the scratch-wound assay are shown for the cells transfected with the *Mgp* shRNA or control shRNA and treated with TGFβ. b) Representative images of the scratch-wound assay are shown for the cells treated with TGFβ and increasing concentrations of MGP.

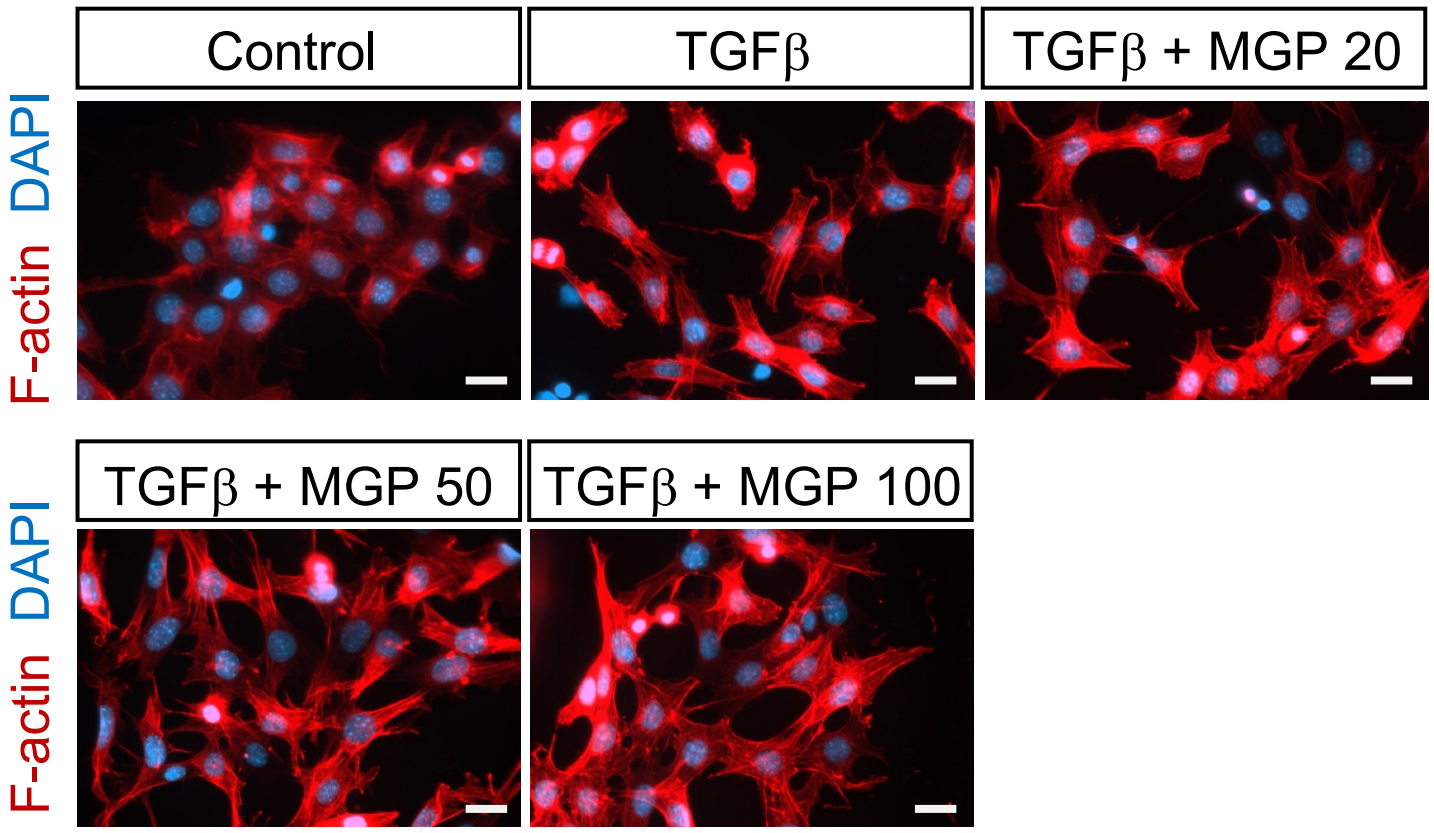

Sup Fig 15. Effect of TGFβ and MGP on cytoskeletal reorganization in NIH3T3 cells. Phalloidin-Rhodamine staining was performed to assess for stress fiber formation. Scale bar: 10 μm. Representative images of at least three independent replicates are shown. MGP concentration in ng/mL.

Patient	IFTA	Age	Sex	Pathologist Diagnosis
1	I	66	Male	Banff 2022: Interstitial Fibrosis and Tubular Atrophy Grade I
2	I	61	Male	Banff 2022: Interstitial Fibrosis and Tubular Atrophy Grade I
3	I	48	Male	Banff 2022: Interstitial Fibrosis and Tubular Atrophy Grade I
4	I	42	Male	Banff 2022: Interstitial Fibrosis and Tubular Atrophy Grade I
5	I	65	Male	Banff 2022: Interstitial Fibrosis and Tubular Atrophy Grade I
6	III	28	Female	Calcineurin Toxicity, Banff 2022: Interstitial Fibrosis and Tubular Atrophy Grade III
7	III	32	Female	BK virus nephropathy, Banff 2022: Interstitial Fibrosis and Tubular Atrophy Grade III
8	III	66	Male	Suspected humoral chronic rejection,Banff 2022: Interstitial Fibrosis and Tubular Atrophy Grade III
9	III	57	Female	BK virus nephropathy, Banff 2022: Interstitial Fibrosis and Tubular Atrophy Grade III
10	III	58	Female	BK virus nephropathy, Banff 2022: Interstitial Fibrosis and Tubular Atrophy Grade III

Sup Table 1. Clinical characteristics for kidney transplant patients at the time of kidney biopsy. IFTA = Interstitial Fibrosis and Tubular Atrophy
